# Saturable Absorption of Free-Electron Laser Radiation by Graphite near the Carbon K-Edge

**DOI:** 10.1021/acs.jpclett.2c01020

**Published:** 2022-09-27

**Authors:** Lars Hoffmann, Sasawat Jamnuch, Craig P. Schwartz, Tobias Helk, Sumana L. Raj, Hikaru Mizuno, Riccardo Mincigrucci, Laura Foglia, Emiliano Principi, Richard J. Saykally, Walter S. Drisdell, Shervin Fatehi, Tod A. Pascal, Michael Zuerch

**Affiliations:** † Department of Chemistry, University of California, Berkeley, California 94720, United States; ‡ Chemical Sciences Division, Lawrence Berkeley National Laboratory, Berkeley, California 94720, United States; § Fritz Haber Institute of the Max Planck Society, 14195 Berlin, Germany; ∥ ATLAS Materials Science Laboratory, Department of Nano Engineering and Chemical Engineering, University of California San Diego, La Jolla, California 92023, United States; ⊥ Nevada Extreme Conditions Laboratory, University of Nevada, Las Vegas, Las Vegas, Nevada 89154, United States; # Institute of Optics and Quantum Electronics, Abbe Center of Photonics, Friedrich-Schiller University, 07743 Jena, Germany; ∇ Helmholtz Institute Jena, 07743 Jena, Germany; ⊗ Elettra-Sincrotrone Trieste S.C.p.A., Strada Statale 14, 34149 Trieste, Italy; × Joint Center for Artificial Photosynthesis, Lawrence Berkeley National Laboratory, Berkeley, California 94720, United States; ☆ Department of Chemistry, The University of Texas Rio Grande Valley, Edinburg, Texas 78539, United States; △ Materials Science and Engineering, University of California San Diego, La Jolla, California 92023, United States; ▼ Sustainable Power and Energy Center, University of California San Diego, La Jolla, California 92023, United States; ▲ Materials Sciences Division, Lawrence Berkeley National Laboratory, Berkeley, California 94720, United States

## Abstract

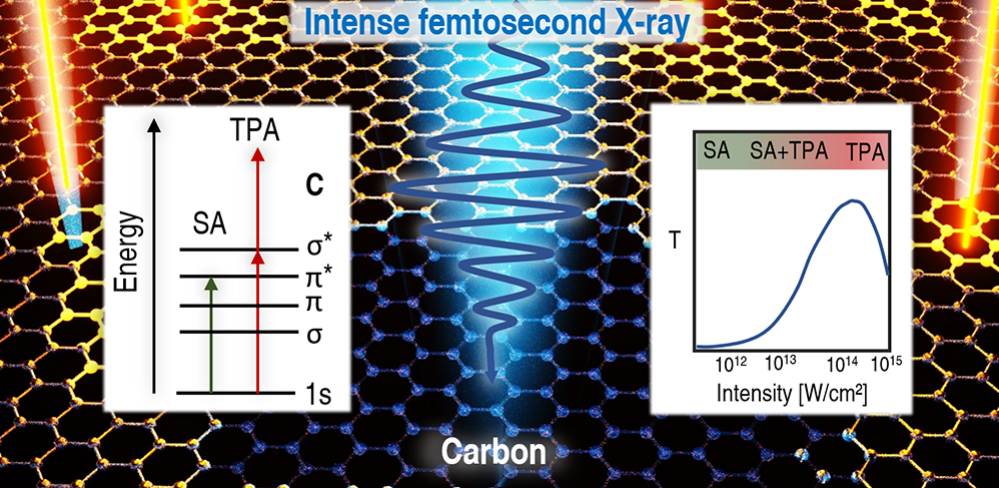

The interaction of intense light with matter gives rise to competing nonlinear responses that can dynamically change material properties. Prominent examples are saturable absorption (SA) and two-photon absorption (TPA), which dynamically increase and decrease the transmission of a sample depending on pulse intensity, respectively. The availability of intense soft X-ray pulses from free-electron lasers (FELs) has led to observations of SA and TPA in separate experiments, leaving open questions about the possible interplay between and relative strength of the two phenomena. Here, we systematically study both phenomena in one experiment by exposing graphite films to soft X-ray FEL pulses of varying intensity. By applying real-time electronic structure calculations, we find that for lower intensities the nonlinear contribution to the absorption is dominated by SA attributed to ground-state depletion; our model suggests that TPA becomes more dominant for larger intensities (>10^14^ W/cm^2^). Our results demonstrate an approach of general utility for interpreting FEL spectroscopies.

Saturable absorption (SA) is a nonlinear optical response characterized by a reduction in the relative absorption of the sample—or, conversely, an increase in transmission—with an increase in the intensity of incident light. Although this effect has been known since the 1940s,^1^ direct experimental observation has been limited due to the high intensity required of the incident light, which was unachievable before the development of modern laser technology. The phenomenon has now been widely demonstrated and applied in the visible and infrared regions.^2^ Moreover, saturable absorbers play a key role in passive mode-locking of femtosecond laser oscillators.^3,4^ Saturable absorption is exquisitely sensitive to the electronic state of the material being probed. As such, SA is often used as a probe of dynamics and recovery following a pump–probe event, typically using a probe in the visible or infrared. It can be used to extract detailed information on photoexcited scattering and diffusion of charge carriers, as well as structural changes such as recovery following melting.^5,6^

As compared with studying SA in transitions between electronic valence states, which may exhibit significant hybridization, studying SA in transitions from the well-localized and minimally hybridized core to valence states provides a more direct route to understanding the underlying processes. Such studies became possible with the advent of free-electron lasers (FELs), which combine very high intensities with high photon energies to enable excitations resonant with, and capable of depleting, a chosen ground state in the XUV to hard X-ray regions. At the FLASH FEL, SA of the L-shell transition of aluminum was observed with photon energies of 92 eV (13 nm).^7^ Subsequent observations have been reported for the tin N-edge at 24 eV (52 nm) and for the iron K-edge at 7.1 keV (0.17 nm).^8,9^ In these experiments, SA was attributed to high photon flux depleting the ground state, which led to X-ray-induced transparency. The intensity at which this transparency occurs is dependent on the energy of the absorption edge. Deeper lying electrons result in core-holes with shorter lifetime, which increases the intensity that is necessary to observe SA.

In similar experimental settings, by contrast, unusually high absorption has been observed at the aluminum K-edge at 1560 eV (0.79 nm) and carbon K-edge at 285 eV (4.35 nm), which was attributed to two-photon absorption (TPA).^10,11^ Both observations of TPA used photon energies close to absorption edges where TPA is enhanced by resonance effects. Stöhr and Scherz separately proposed that X-ray induced transparency could be induced by stimulated elastic forward scattering, and they worked out the consequences of this mechanism in order to explain the properties of metallic cobalt at the L_3_-edge.^12^ Further support for this hypothesis was provided by experiments on Co/Pd multilayers, where a strong flux dependence was observed in the resulting transmission and diffraction contrast. As the flux increased, transmission increased and diffraction contrast decreased; this trend was ascribed to stimulated processes, including stimulated emission.^13^ The loss of diffraction was consistent with that observed previously in Co/Pd multilayers.^14^ These contrasting observations and explanations demonstrate that a complete physical picture of the processes at play when high-intensity X-ray pulses interact with solid-state systems is still missing. In addition, because core-hole lifetimes are typically on the order of femtoseconds,^15^ comparable to the pulse duration of X-ray FELs, these are transient phenomena exhibiting a strong dependence on the pulse duration.

Here we employ femtosecond FEL light to systematically study the intensity dependence of SA in graphite around the carbon K-edge, in the soft X-ray region. We target several transition energies to excite the carbon 1s core electrons into different valence states. Moreover, time-dependent density functional theory (TDDFT) simulations are used to show that the experimental measurements contain signatures of both SA and TPA, with TPA becoming more dominant at higher intensities. While SA and TPA have been observed before, to our knowledge, this work is the first to observe both regimes in one experiment and to disentangle the respective contributions. To separately account for SA and TPA is especially important for interpreting X-ray spectra taken in intensity regimes where both phenomena make significant contributions.

The experiments were performed at the EIS-TIMEX beamline of the FERMI FEL.^16−18^ The FEL beam first passed through the Photon Analysis, Delivery, and Reduction System (PADReS), which includes beam diagnostics and provides the incident pulse energy for every FEL shot. The FEL pulse (photon energy = 285.7–309.2 eV, pulse duration τ ≈ 25 fs fwhm, pulse energy *E*_p_ = 4–18 μJ, spot size 12 × 12 μm^2^) was focused on the sample (Figure 1a). The FEL was set to maximum intensity for each photon energy which resulted in higher maximum intensity for the lower energy of 285.7 eV. The transmitted beam was propagated through a 50 nm Ni filter to prevent camera saturation, then dispersed by a spherical diffraction grating (HZB 1603 2, Au reflection coated, 1000 ± 2 gr/mm, 11 ± 1.5 nm groove depth, 0.6 ± 0.1 nm groove spacing, manufactured by Helmholtz Zentrum Berlin) onto a CCD camera (Andor iKon-M SO). A single spectrum was recorded for each pulse. The samples were unsupported films of polycrystalline graphite with thickness of 80 ± 8 nm. Because the FEL pulses caused sample damage, the films were raster scanned to probe a pristine spot with each shot. For each laser shot, comparing the recorded incident photon flux with the recorded photon flux post-sample results in a measure of sample transmission as a function of incident flux. The observed transmission is subsequently compared to the linear Beer–Lambert law. Use of the Beer–Lambert law assumes negligible reflectivity at normal incidence, which holds for carbon at 300 eV (refractive index *n* ≈ 1).^19^ The FEL photon energy is varied to probe π* (285.7 eV) and σ* (309.2 eV) regions from the 1s core state. Knowledge of the pulse envelope is essential for correctly determining the pulse intensity; the seeded FERMI FEL is known to deliver pulses with a Gaussian envelope, similar to the sin^2^ envelopes commonly used in theoretical calculations.^20^

**Figure 1:**
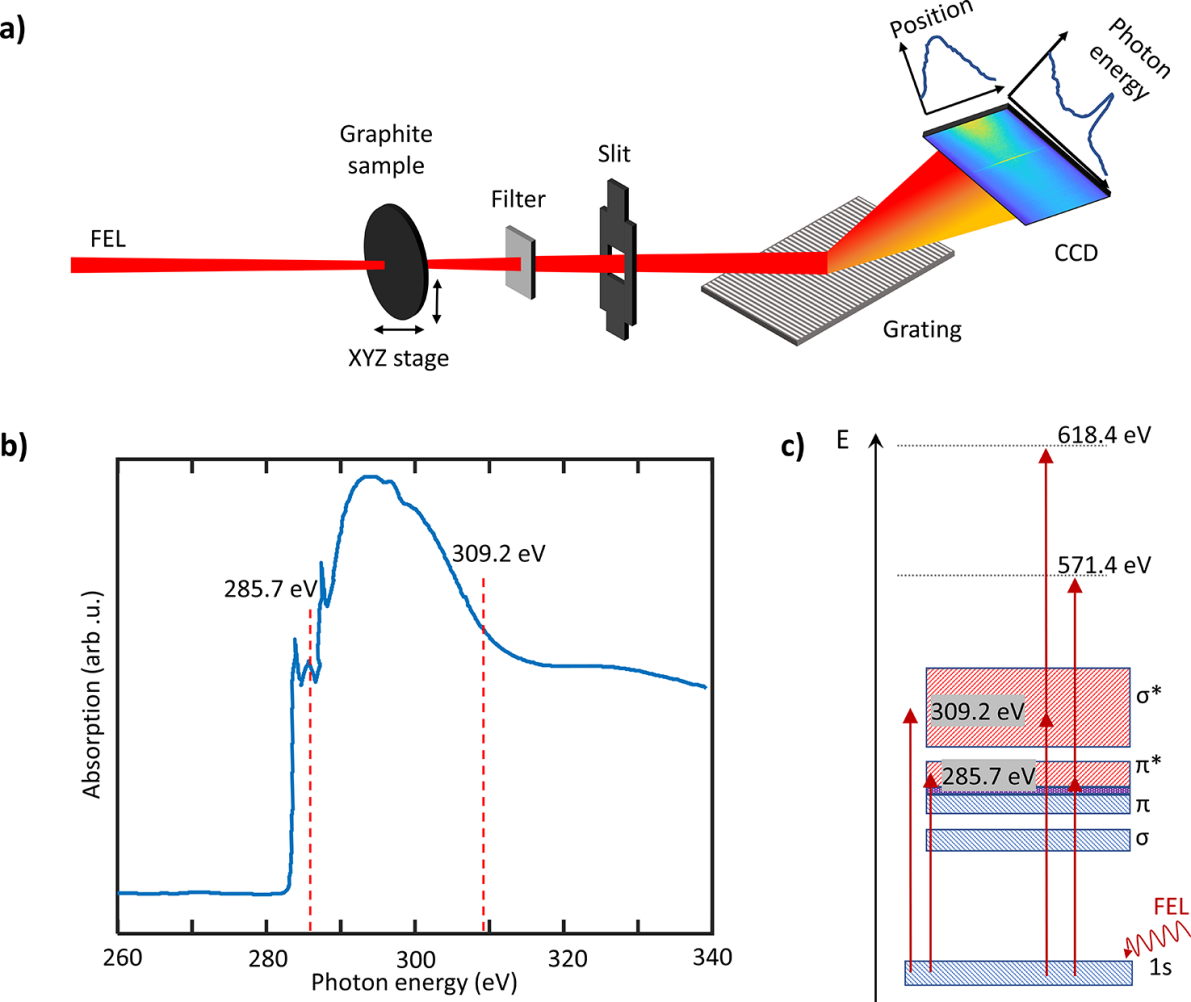
Experimental setup and transitions in graphite. (a) Tunable FEL pulses were focused on the graphite sample. Ni metal foil was used to filter prevent camera saturation. An imaging spectrometer collected the transmitted X-ray light. To reference the incoming X-ray pulse intensity, a gas ionization−based intensity monitor was used downstream. (b) X-ray absorption spectrum of a 500 nm graphite sample (data taken from ref (11)). X-ray transmission measurements were conducted at discrete photon energies indicated with dashed lines. (c) Energy-level diagram of the absorption at the absorption edge in resonance with the π*-orbital (285.7 eV) and above the edge in resonance with the σ*-orbital (309.2 eV) as well as the respective two-photon absorptions. The dark blue box between π and π* represents the Fermi energy.

For a mechanistic understanding of the experiment, velocity-gauge real-time time-dependent density functional theory (VG-RTTDDFT) was employed^21,22^ with a numerical atomic orbital basis set in order to propagate the electronic structure of graphite under an intense laser field. Exchange-correlation (XC)^23^ effects are treated within the adiabatic local density approximation using the Perdew–Zunger LDA.^24^ The carbon pseudopotential was generated by pseudoizing C:{1s,2p,3d} with the explicit inclusion of a 1s core-hole. The C 2s state was then obtained as a higher energy solution of the atomic Schrödinger equation. The graphite primitive unit cell was subsequently propagated for 25 fs under a sin^2^-enveloped pulse centered at *t* = 12.5 fs, with incident pulse intensities ranging from 10^10^ to 10^14^ W/cm^2^. The calculations were performed for photon energies corresponding to experimental values for the π* and σ* regions.

The excitation scheme is illustrated in Figure 1a, with the linear X-ray absorption spectrum of graphite^25^ shown in Figure 1b as a reference for the FEL energies used in this study. We note that the previously measured spectrum was taken from a 500 nm thick graphite foil. While we expect the thickness to influence the nonlinear absorption characteristics, the spectrum should be comparable to our 80 nm sample. At the selected energies, the FEL excites the core electrons into either the π* orbitals or the σ* orbitals. By tuning the FEL to two different electronic transitions of graphite we can make use of the different absorption properties of the 1s-to-π* and 1s-to-σ* transitions. The corresponding energy-level diagram is shown in Figure 1c, indicating the respective transitions. The transmitted X-ray flux was measured as a function of varying FEL intensity, and it was observed that the transmitted X-ray intensity increases with the incoming intensity in a strongly nonlinear fashion. Figure 2a,b shows the transmission through the sample at a range of intensities and for two specific energies. In Figure 2a, probing 1s-to-π* transitions, the transmitted intensity is lower than expected relative to the linear absorption behavior modeled by the Beer–Lambert law, i.e., sub-linear. This effect, also known as “reverse” SA, has been reported for the aluminum K-edge and carbon K-edge and was attributed to TPA.^10,11^ In Figure 2b, probing 1s-to-σ* transitions, the transmitted intensity is greater than expected from a linear response, i.e., super-linear. This nonlinear increase in transmitted intensity mirrors the previously reported observation of SA for the aluminum L-edge and iron K-edge.^7,9^ In those cases, SA is caused by the finite core-hole lifetime, which at soft X-ray energies depends mainly on the rate of Auger decay. For graphite, the lifetime of the 1s hole was calculated to be 7 fs,^26^ which is of the same order of magnitude as the FEL pulse duration (∼25 fs). We point out that Figure 2a,b plots the transmitted X-ray intensity itself, while Figure 2c,d plots the transmitted intensity as a fraction of the incoming intensity. Consequently, a linear trend in Figure 2a,b corresponds to a constant trend in Figure 2c,d. A more direct comparison of our calculated results with the experimental transmission would require calibration of the FEL intensity downstream of the sample, which is not available. The absence of this calibration neither limits our ability to observe nonlinear effects (by comparison with a linear fit) nor alters the trends we observed at each wavelength.

**Figure 2:**
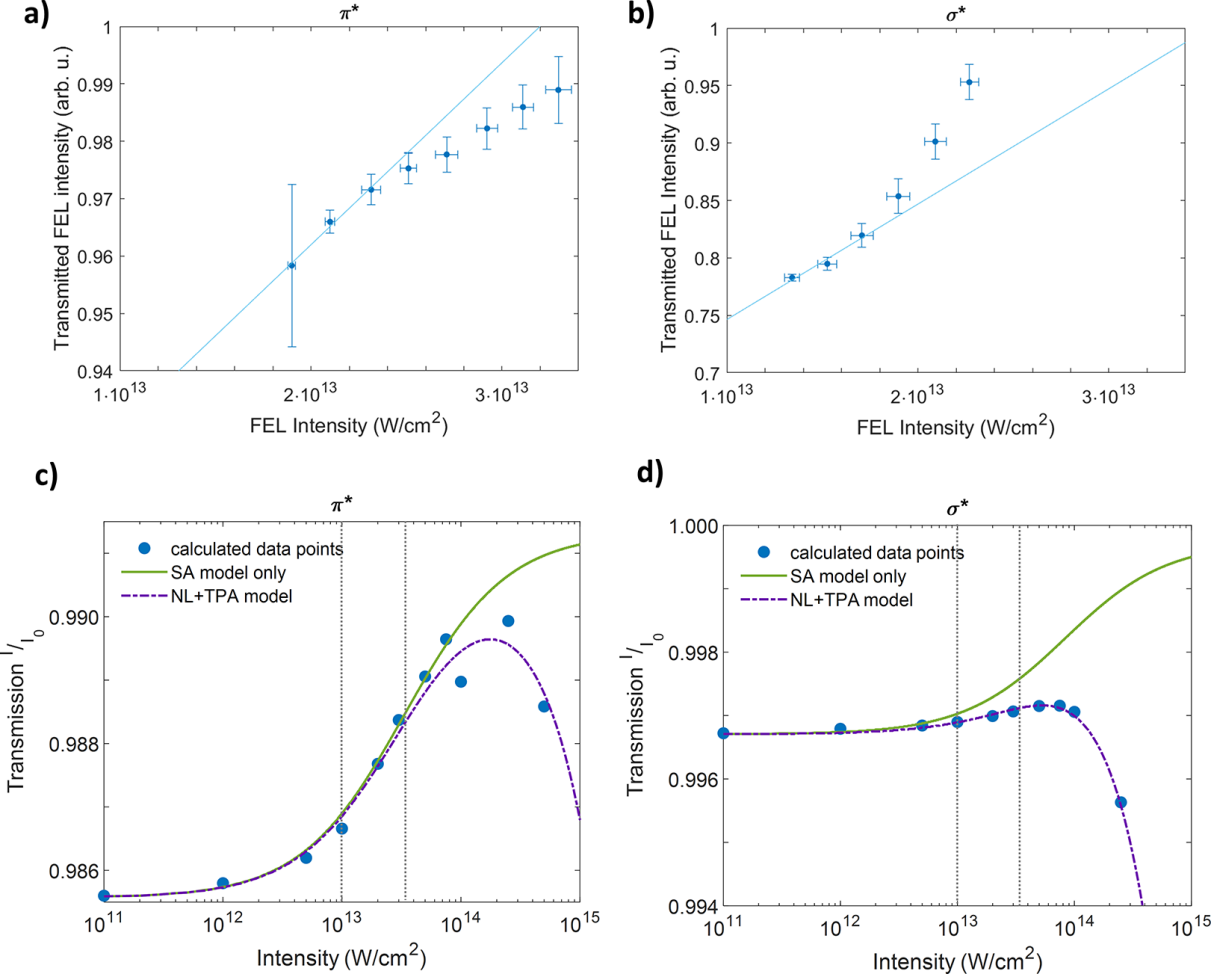
Experimental and simulated transmission for resonant excitation from C 1s into π* and σ* states. (a, b) Experimental transmitted intensity for π* (285.7 eV) and σ* (309.2 eV) for incoming FEL intensities in the regions shown in (c) and (d) as dotted lines. Plot (a) shows an increase in absorption, while plot (b) shows a decrease in absorption relative to a linear absorption model. Respective linear fits to the first three data points highlight the differing nonlinear behavior. (c, d) Simulated transmission vs intensity for π* and σ* fitted to the model in eq 1, including two-photon absorption (TPA, purple dashed line). Comparison with a model neglecting TPA (green line) shows that TPA becomes dominant at high intensities. Fluctuations in panel c are due to competition between the TPA process and relaxation of excited electrons; the σ* does not exhibit this behavior due to a stronger TPA response. Gray dotted lines indicate the intensity range that was measured in the experiment. Note that (a) and (b) show the transmitted intensity, while (c) and (d) show the transmitted intensity as a fraction of the incoming intensity.

To gain additional insights into the observed trends, we turn our attention to first-principles calculations. In these calculations, the time evolution of the absorbed energy per unit input, *E*_absorbed_(*t*) = *E*(*t*) – *E*(0), is evaluated at two different photon energies, representing the 1s-to-π* and -σ* transitions. We note that the energy deposited into the system is conserved. Additional calculations for the pre-edge can be found in the Supporting Information, Figure S1.^27^ For π* transitions (Figure 3a), a clear trend indicative of SA is visible in the range of 10^11^–10^14^ W/cm^2^, with the absorbed energy decreasing as the intensity increases. Transitions at and above an intensity of 10^14^ W/cm^2^ do not uniformly follow this trend, sometimes showing increasing absorption. For the σ* transitions (Figure 3b), similar behavior indicative of SA is observed for intensities of 10^11^–10^13^ W/cm^2^. Around 10^14^ W/cm^2^, a drastically different behavior is observed in the calculation, with the absorption increasing substantially. The Fourier transform of the time-dependent current at 10^14^ W/cm^2^ (Figure 3c,d) shows an emerging signal at 2ω, indicating TPA. In addition, the overall absorption increases significantly for approximately 10 fs after excitation onset, then levels off after another 5–10 fs. The plateau suggests a core-depletion effect leading to two-photon excitations to very high energy states. To test this hypothesis, we evaluated the final energy of the system at *t* = 25 fs, i.e., the total energy absorbed from the X-ray pulse. The transmitted intensity inferred from the energy absorbed by the system was fit to a model allowing for contributions from saturable, non-saturable, and two-photon absorption:
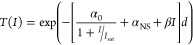
1

**Figure 3:**
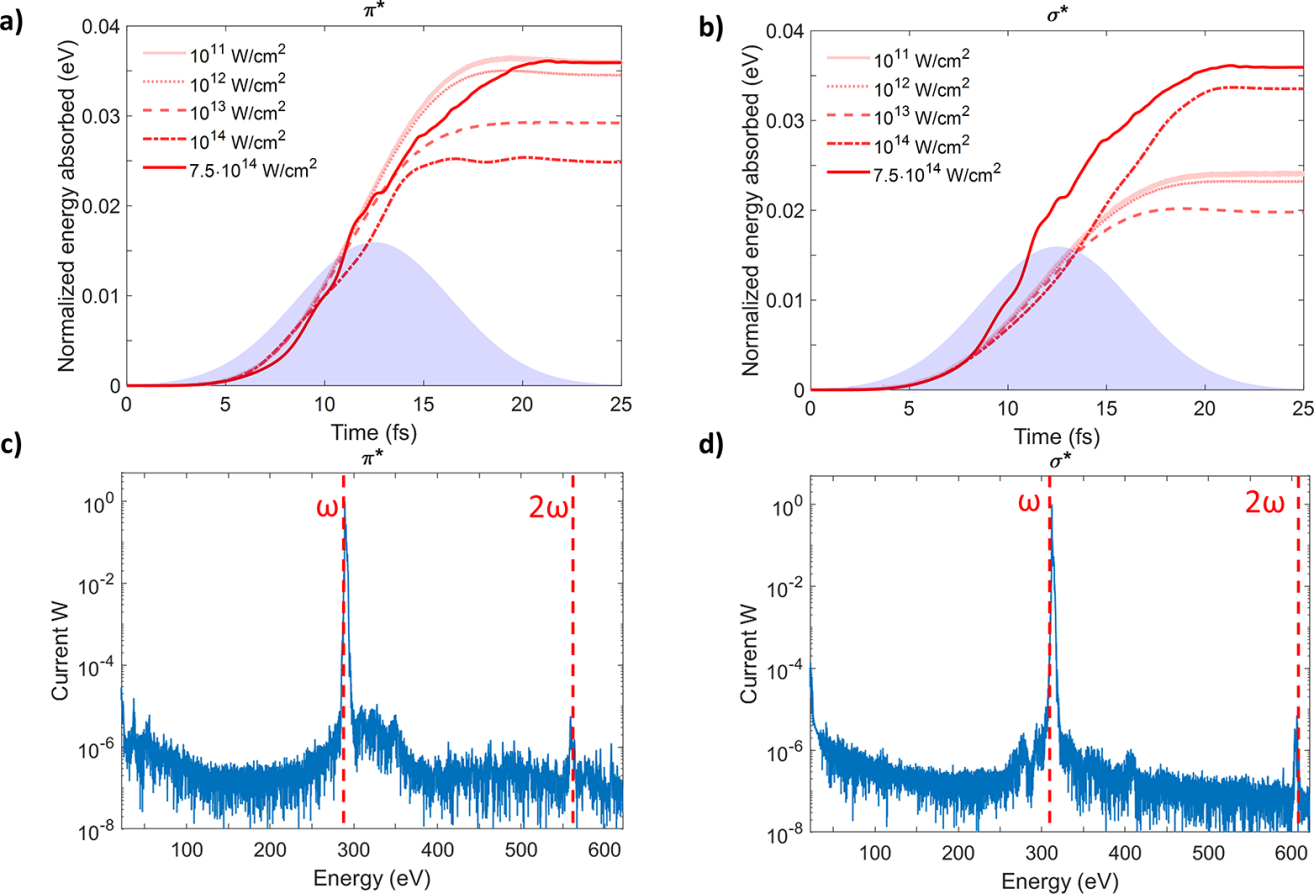
Simulation of time-dependent absorption for resonant excitation from C 1s into π* and σ*. (a, b) Time-dependent absorption for the transitions C 1s→π* (285.7 eV) and C 1s→σ* (309.2 eV) for different X-ray intensities, respectively. The purple shaded area represents the envelope of the driving pulse used for the numerical simulation. Shown in (c) and (d) are the corresponding normalized Fourier transforms of the time evolution of the current at 10^14^ W/cm^2^, indicating an additional component of the absorption at the highest intensities that stems from absorption of two photons of the FEL pulse 2ω. Both curves (c) and (d) are normalized to their maximum peak amplitude.

Here *T(I)* is the transmission; α_0_, α_NS_, and β are constants related to saturable, non-saturable, and two-photon absorption, respectively; and *I*_sat_ is a characteristic saturation intensity. The thickness *d* is set to 0.68 nm, matching the thickness of the graphite primitive cell used in the simulation. The thickness of the calculated graphite model increases the total transmission relative to that of the experimental sample (80 nm) but does not change the line shape. As shown in Figures 2c,d, this model (purple dashed line) produces good fits to the calculated intensity data (solid blue circles) for both the 1s-to-π* and -σ* transitions (*R*^2^ = 0.98 and 0.96, respectively; see Supporting Information, Table S1 for the parameter values).^27^ If TPA is omitted (green line in Figures 2c,d), however, a monotonic increase in transmission is observed. Our model indicates, then, that the increase in transmission at relatively low intensity is purely due to SA. At higher intensities, ratios of the model constants (Table S1) show that SA is approximately twice as strong at the 1s-to-π* transition compared to the 1s-to-σ* transition, and TPA is approximately 3 times stronger at the σ*.

Auger decay events—and any sequential absorption processes following the formation of the resulting valence hole^28,29^—are not captured by the level of theory applied here. That said, Auger decay is unlikely to have played a significant role in these experiments due to the narrow spectral width and high spectral stability of the FEL source tuned into resonance with the 1s-to-π* and 1s-to-σ* transitions. A simulated linear XAS spectrum for the same type of material exhibits the 1s-to-π* and 1s-to-σ* transitions for which the FEL experiments were performed (see Supporting Information, Figure S2). Therefore, any energy-level shifts arising from core-hole effects or secondary excitations such as Auger decay should be captured at least qualitatively correctly. Some uncertainty remains due to the femtosecond nature of the photoexcitation pulse, which may entail a slightly different balance of secondary excitation events than in synchrotron-based XAS; sub-femtosecond screening effects, however, should be largely independent of the time scales present in the experiment.^30,31^ Another possible source of inconsistencies between the experiment and simulations of the TPA process is the varying thickness and structuring of the sample. In the simulation, we assume a crystalline graphite sample oriented such that the polarization of the electric field of the FEL is perpendicular to the graphite layer (i.e., along the *c*-axis) for the π* excitation and parallel to it (i.e., in the *ab* plane) for the σ* excitation. This geometry maximizes the overall absorption, which may lead to an overestimation of absorption-induced effects from theory.^32^ In the experiment, by contrast, the sample is polycrystalline and by definition includes randomness in its structure. To gauge the significance of this difference, we simulate the effect of changing the polarization of the incoming photon on the energy absorbed. We observe that the absorption is indeed greatly reduced if the photon’s field of polarization is not well-aligned with the respective excitation in the sample. The simulated linear absorption spectrum, additional details of the numerical simulation parameters, and comparison of absorbed energies as a function of incident photon polarization can be found in the Supporting Information.

With these insights in hand, we can return to consideration of the experiment, and in particular, the sub-linear (TPA-dominated) and super-linear (SA-dominated) behavior of the transmission with increasing intensity at the respective 1s-to-π* and 1s-to-σ* transitions. To explain the difference between π* and σ* transitions, we first note that excitation into the σ* state at normal incidence is known to have a larger oscillator strength than excitation into the π* state.^33^ While the samples used here are polycrystalline rather than single crystals, we expect that a similar reduction in the likelihood of initial core excitation into the π* limits the ground-state depletion that favors SA rather than TPA, thereby reducing the transmitted intensity relative to the linear model. At the 1s-to-σ* transition, by contrast, depletion of the 1s ground state is more likely, and SA can dominate in the measured intensity range. Second, we emphasize that Figure 2a,b covers different intensity ranges. The experimental data for 1s-to-π* transitions in Figure 2a span from 1.9 × 10^13^ to 3.3 × 10^13^ W/cm^2^, while those for 1s-to-σ* transition in Figure 2b cover a more limited and lower intensity range, from 1.3 × 10^13^ to 2.2 × 10^13^ W/cm^2^. Although TPA is calculated to be more likely for the 1s-to-σ* transition than for the 1s-to-π* transition at comparable intensities, the observed difference in behavior can be attributed to the higher FEL intensities achieved during our measurements^11^ for π*. In previous measurements SA has not been observed at 307.86 eV, which is close to the photon energy of 309.2 eV that was applied here. Several parameters of this experiment differ from previous measurements^11^ and could lead to enhancement of SA. In particular, the sample used here was thinner, and the FEL spot size was smaller, which leads to larger measured intensities at similar pulse energies.

To summarize, in this work we report the complex interplay of SA and TPA of intense X-rays in graphite, taking advantage of the high intensities available at EIS-TIMEX to measure transmission near the carbon K-edge. In contrast to the mechanism proposed to explain previous observations of SA in graphite,^11^ our calculations indicate that this trend of increasing transmission with increasing intensity originates in depletion of the core by the intense FEL pulse which persists until a regime is reached where TPA becomes dominant. Data collected at intensities of up to ∼10^13^ W/cm^2^ exhibited a decrease or an increase in transmission with intensity, relative to a linear response, for the respective 1s-to-π* and 1s-to-σ* transitions. We attribute this behavior to different transition dipoles shifting the regime where TPA becomes dominant over SA at different threshold intensities. Our calculations indicate that TPA will dominate for both photon energies at intensities greater than ∼10^14^ W/cm^2^. We expect that improvements in the simulation methodology, including a better description of the electron–hole screening and more extensive sampling of the ground state starting structure to include finite temperature effects, will lead to even better agreement in the predicted intensities, and will be explored in future works. Our experimental methods combined with theoretical calculations enable additional insights into nonlinear processes that occur due to the absorption of intense radiation at X-ray energies and can readily be extended to other materials. These findings are relevant for correctly interpreting X-ray absorption and scattering data collected at high intensities, especially in regimes where both phenomena make significant contributions.

## Supporting Information Available

Computational methods, simulated linear absorption spectrum, calculated time-dependent pre-edge absorption, calculated transmission for 80 nm graphite sample, polarization dependence of the π* absorption, and fitted model parameters (PDF)
Transparent Peer Review report available (PDF)

## References

[ref1] Lewis G. N.; Lipkin D.; Magel T. T. Reversible Photochemical Processes in Rigid Media. A Study of the Phosphorescent State. J. Am. Chem. Soc. 1941, 63 (11), 3005–3018. 10.1021/ja01856a043.

[ref2] Li C. Nonlinear Optics - Principles and Application; Springer Singapore: Singapore, 2016.

[ref3] DeMaria A. J.; Stetser D. A.; Heynau H. Self Mode-locking of Lasers with Saturable Absorbers. Appl. Phys. Lett. 1966, 8 (7), 174–176. 10.1063/1.1754541.

[ref4] Paschotta R.; Keller U. Passive Mode Locking with Slow Saturable Absorbers. Appl. Phys. B: Laser Opt. 2001, 73 (7), 653–662. 10.1007/s003400100726.

[ref5] Wang G.; Baker-Murray A. A.; Zhang X.; Bennett D.; Wang J. J.; Wang J.; Wang K.; Blau W. J. Broadband Saturable Absorption and Exciton-Exciton Annihilation in MoSe_2_ Composite Thin Films. Opt. Mater. Express 2019, 9 (2), 483–496. 10.1364/OME.9.000483.

[ref6] Kumar S.; Anija M.; Kamaraju N.; Vasu K. S.; Subrahmanyam K. S.; Sood A. K.; Rao C. N. R. Femtosecond Carrier Dynamics and Saturable Absorption in Graphene Suspensions. Appl. Phys. Lett. 2009, 95 (19), 191911 10.1063/1.3264964.

[ref7] Nagler B.; Zastrau U.; Fäustlin R. R.; Vinko S. M.; Whitcher T.; Nelson A. J.; Sobierajski R.; Krzywinski J.; Chalupsky J.; Abreu E.; Bajt S.; Bornath T.; Burian T.; Chapman H.; Cihelka J.; Döppner T.; Düsterer S.; Dzelzainis T.; Fajardo M.; Förster E.; Fortmann C.; Galtier E.; Glenzer S. H.; Göde S.; Gregori G.; Hajkova V.; Heimann P.; Juha L.; Jurek M.; Khattak F. Y.; Khorsand A. R.; Klinger D.; Kozlova M.; Laarmann T.; Lee H. J.; Lee R. W.; Meiwes-Broer K.-H.; Mercere P.; Murphy W. J.; Przystawik A.; Redmer R.; Reinholz H.; Riley D.; Röpke G.; Rosmej F.; Saksl K.; Schott R.; Thiele R.; Tiggesbäumker J.; Toleikis S.; Tschentscher T.; Uschmann I.; Vollmer H. J.; Wark J. S. Turning Solid Aluminium Transparent by Intense Soft X-Ray Photoionization. Nat. Phys. 2009, 5 (9), 693–696. 10.1038/nphys1341.

[ref8] Yoneda H.; Inubushi Y.; Tanaka T.; Yamaguchi Y.; Sato F.; Morimoto S.; Kumagai T.; Nagasono M.; Higashiya A.; Yabashi M.; Ishikawa T.; Ohashi H.; Kimura H.; Kitamura H.; Kodama R. Ultra-Fast Switching of Light by Absorption Saturation in Vacuum Ultra-Violet Region. Opt. Express 2009, 17 (26), 23443 10.1364/OE.17.023443. 20052051

[ref9] Yoneda H.; Inubushi Y.; Yabashi M.; Katayama T.; Ishikawa T.; Ohashi H.; Yumoto H.; Yamauchi K.; Mimura H.; Kitamura H. Saturable Absorption of Intense Hard X-Rays in Iron. Nat. Commun. 2014, 5 (1), 5080 10.1038/ncomms6080. 25270525

[ref10] Cho B. I.; Cho M. S.; Kim M.; Chung H.-K.; Barbrel B.; Engelhorn K.; Burian T.; Chalupský J.; Ciricosta O.; Dakovski G. L.; Hájková V.; Holmes M.; Juha L.; Krzywinski J.; Lee R. W.; Nam C. H.; Rackstraw D. S.; Toleikis S.; Turner J. J.; Vinko S. M.; Wark J. S.; Zastrau U.; Heimann P. A. Observation of Reverse Saturable Absorption of an X-Ray Laser. Phys. Rev. Lett. 2017, 119 (7), 075002 10.1103/PhysRevLett.119.075002. 28949680

[ref11] Lam R. K.; Raj S. L.; Pascal T. A.; Pemmaraju C. D.; Foglia L.; Simoncig A.; Fabris N.; Miotti P.; Hull C. J.; Rizzuto A. M.; Smith J. W.; Mincigrucci R.; Masciovecchio C.; Gessini A.; De Ninno G.; Diviacco B.; Roussel E.; Spampinati S.; Penco G.; Di Mitri S.; Trovò M.; Danailov M. B.; Christensen S. T.; Sokaras D.; Weng T.-C.; Coreno M.; Poletto L.; Drisdell W. S.; Prendergast D.; Giannessi L.; Principi E.; Nordlund D.; Saykally R. J.; Schwartz C. P. Two-Photon Absorption of Soft X-Ray Free Electron Laser Radiation by Graphite near the Carbon K-Absorption Edge. Chem. Phys. Lett. 2018, 703, 112–116. 10.1016/j.cplett.2018.05.021.

[ref12] Stöhr J.; Scherz A. Creation of X-Ray Transparency of Matter by Stimulated Elastic Forward Scattering. Phys. Rev. Lett. 2015, 115 (10), 107402 10.1103/PhysRevLett.115.107402. 26382702

[ref13] Chen Z.; Higley D. J.; Beye M.; Hantschmann M.; Mehta V.; Hellwig O.; Mitra A.; Bonetti S.; Bucher M.; Carron S.; Chase T.; Jal E.; Kukreja R.; Liu T.; Reid A. H.; Dakovski G. L.; Föhlisch A.; Schlotter W. F.; Dürr H. A.; Stöhr J. Ultrafast Self-Induced X-Ray Transparency and Loss of Magnetic Diffraction. Phys. Rev. Lett. 2018, 121 (13), 137403 10.1103/PhysRevLett.121.137403. 30312105

[ref14] Wu B.; Wang T.; Graves C. E.; Zhu D.; Schlotter W. F.; Turner J. J.; Hellwig O.; Chen Z.; Dürr H. A.; Scherz A.; Stöhr J. Elimination of X-Ray Diffraction through Stimulated X-Ray Transmission. Phys. Rev. Lett. 2016, 117 (2), 027401 10.1103/PhysRevLett.117.027401. 27447522

[ref15] Krause M. O.; Oliver J. H. Natural Widths of Atomic K and L Levels, Kα X-ray Lines and Several KLL Auger Lines. J. Phys. Chem. Ref. Data 1979, 8 (2), 329–338. 10.1063/1.555595.

[ref16] Allaria E.; Callegari C.; Cocco D.; Fawley W. M.; Kiskinova M.; Masciovecchio C.; Parmigiani F. The FERMI@Elettra Free-Electron-Laser Source for Coherent x-Ray Physics: Photon Properties, Beam Transport System and Applications. New J. Phys. 2010, 12 (7), 075002 10.1088/1367-2630/12/7/075002.

[ref17] Masciovecchio C.; Battistoni A.; Giangrisostomi E.; Bencivenga F.; Principi E.; Mincigrucci R.; Cucini R.; Gessini A.; D’Amico F.; Borghes R.; Prica M.; Chenda V.; Scarcia M.; Gaio G.; Kurdi G.; Demidovich A.; Danailov M. B.; Di Cicco A.; Filipponi A.; Gunnella R.; Hatada K.; Mahne N.; Raimondi L.; Svetina C.; Godnig R.; Abrami A.; Zangrando M. EIS: The Scattering Beamline at FERMI. J. Synchrotron Radiat. 2015, 22 (3), 553–564. 10.1107/S1600577515003380. 25931068

[ref18] Giannessi L.; Allaria E.; Badano L.; Bencivenga F.; Callegari C.; Capotondi F.; Cilento F.; Cinquegrana P.; Coreno M.; Cudin I.; D’Auria G.; Danailov M.; De Monte R.; De Ninno G.; Delgiusto P.; Demidovich A.; Di Fraia M.; Di Mitri S.; Diviacco B.; Fabris A.; Fabris R.; Fawley W.; Ferianis M.; Furlan Radivo P.; Gaio G.; Gauthier D.; Gelmetti F.; Iazzourene F.; Krecic S.; Lonza M.; Mahne N.; Malvestuto M.; Masciovecchio C.; Milloch M.; Mirian N.; Parmigiani F.; Penco G.; Perucchi A.; Pivetta L.; Plekan O.; Predonzani M.; Principi E.; Raimondi L.; Rebernik Ribič P.; Rossi F.; Roussel E.; Rumiz L.; Scafuri C.; Serpico C.; Sigalotti P.; Spampinati S.; Spezzani C.; Svandrlik M.; Trovò M.; Vascotto A.; Veronese M.; Visintini R.; Zangrando D.; Zangrando M. Status and Perspectives of the FERMI FEL Facility. Proc. 38th Int. Free Electron Laser Conf. 2018, FEL2017 10.18429/JACoW-FEL2017-MOD04.

[ref19] Principi E.; Krylow S.; Garcia M. E.; Simoncig A.; Foglia L.; Mincigrucci R.; Kurdi G.; Gessini A.; Bencivenga F.; Giglia A.; Nannarone S.; Masciovecchio C. Atomic and Electronic Structure of Solid-Density Liquid Carbon. Phys. Rev. Lett. 2020, 125, 155703 10.1103/PhysRevLett.125.155703. 33095640

[ref20] De Ninno G.; Gauthier D.; Mahieu B.; Ribič P. R.; Allaria E.; Cinquegrana P.; Danailov M. B.; Demidovich A.; Ferrari E.; Giannessi L.; Penco G.; Sigalotti P.; Stupar M. Single-Shot Spectro-Temporal Characterization of XUV Pulses from a Seeded Free-Electron Laser. Nat. Commun. 2015, 6 (1), 8075 10.1038/ncomms9075. 26290320 PMC4560797

[ref21] Pemmaraju C. D. Valence and Core Excitons in Solids from Velocity-Gauge Real-Time TDDFT with Range-Separated Hybrid Functionals: An LCAO Approach. Comput. Condens. Matter 2019, 18, e00348 10.1016/j.cocom.2018.e00348.

[ref22] Pemmaraju C. D.; Vila F. D.; Kas J. J.; Sato S. A.; Rehr J. J.; Yabana K.; Prendergast D. Velocity-Gauge Real-Time TDDFT within a Numerical Atomic Orbital Basis Set. Comput. Phys. Commun. 2018, 226, 30–38. 10.1016/j.cpc.2018.01.013.

[ref23] Petersilka M.; Gossmann U. J.; Gross E. K. U. Excitation Energies from Time-Dependent Density-Functional Theory. Phys. Rev. Lett. 1996, 76 (8), 1212–1215. 10.1103/PhysRevLett.76.1212. 10061664

[ref24] Perdew J. P.; Zunger A. Self-Interaction Correction to Density-Functional Approximations for Many-Electron Systems. Phys. Rev. B 1981, 23 (10), 5048–5079. 10.1103/PhysRevB.23.5048.

[ref25] Lam R. K.; Raj S. L.; Pascal T. A.; Pemmaraju C. D.; Foglia L.; Simoncig A.; Fabris N.; Miotti P.; Hull C. J.; Rizzuto A. M.; Smith J. W.; Mincigrucci R.; Masciovecchio C.; Gessini A.; Allaria E.; De Ninno G.; Diviacco B.; Roussel E.; Spampinati S.; Penco G.; Di Mitri S.; Trovò M.; Danailov M.; Christensen S. T.; Sokaras D.; Weng T.-C.; Coreno M.; Poletto L.; Drisdell W. S.; Prendergast D.; Giannessi L.; Principi E.; Nordlund D.; Saykally R. J.; Schwartz C. P. Soft X-Ray Second Harmonic Generation as an Interfacial Probe. Phys. Rev. Lett. 2018, 120 (2), 023901 10.1103/PhysRevLett.120.023901. 29376703

[ref26] Ahuja R.; Brühwiler P. A.; Wills J. M.; Johansson B.; Mårtensson N.; Eriksson O. Theoretical and Experimental Study of the Graphite 1s X-Ray Absorption Edges. Phys. Rev. B 1996, 54 (20), 14396–14404. 10.1103/PhysRevB.54.14396. 9985445

[ref27] See the Supporting Information.

[ref28] Young L.; Kanter E. P.; Krässig B.; Li Y.; March A. M.; Pratt S. T.; Santra R.; Southworth S. H.; Rohringer N.; DiMauro L. F.; Doumy G.; Roedig C. A.; Berrah N.; Fang L.; Hoener M.; Bucksbaum P. H.; Cryan J. P.; Ghimire S.; Glownia J. M.; Reis D. A.; Bozek J. D.; Bostedt C.; Messerschmidt M. Femtosecond Electronic Response of Atoms to Ultra-Intense X-Rays. Nature 2010, 466 (7302), 56–61. 10.1038/nature09177. 20596013

[ref29] Doumy G.; Roedig C.; Son S.-K.; Blaga C. I.; DiChiara A. D.; Santra R.; Berrah N.; Bostedt C.; Bozek J. D.; Bucksbaum P. H.; Cryan J. P.; Fang L.; Ghimire S.; Glownia J. M.; Hoener M.; Kanter E. P.; Krässig B.; Kuebel M.; Messerschmidt M.; Paulus G. G.; Reis D. A.; Rohringer N.; Young L.; Agostini P.; DiMauro L. F. Nonlinear Atomic Response to Intense Ultrashort X Rays. Phys. Rev. Lett. 2011, 106 (8), 083002 10.1103/PhysRevLett.106.083002. 21405568

[ref30] Kanter E. P.; Krässig B.; Li Y.; March A. M.; Ho P.; Rohringer N.; Santra R.; Southworth S. H.; DiMauro L. F.; Doumy G.; Roedig C. A.; Berrah N.; Fang L.; Hoener M.; Bucksbaum P. H.; Ghimire S.; Reis D. A.; Bozek J. D.; Bostedt C.; Messerschmidt M.; Young L. Unveiling and Driving Hidden Resonances with High-Fluence, High-Intensity X-Ray Pulses. Phys. Rev. Lett. 2011, 107 (23), 233001 10.1103/PhysRevLett.107.233001. 22182083

[ref31] Rohringer N.; Santra R. Resonant Auger Effect at High X-Ray Intensity. Phys. Rev. A 2008, 77 (5), 053404 10.1103/PhysRevA.77.053404.

[ref32] Kurata H.; Ishizuka K.; Kobayashi T.; Uyeda N. Orientation Dependence of the Carbon K-Edge in the Electron Energy Loss Spectra of a Potassium-Benzenegraphite Intercalation Compound. Synth. Met. 1988, 22 (4), 337–348. 10.1016/0379-6779(88)90105-1.

[ref33] Buades B.; Moonshiram D.; Sidiropoulos T. P. H.; León I.; Schmidt P.; Pi I.; Di Palo N.; Cousin S. L.; Picón A.; Koppens F.; Biegert J. Dispersive Soft X-Ray Absorption Fine-Structure Spectroscopy in Graphite with an Attosecond Pulse. Optica 2018, 5 (5), 502 10.1364/OPTICA.5.000502.

