# Supplementary material for: Saturable Absorption of Free-Electron Laser Radiation by Graphite near the Carbon K-Edge
Source: J Phys Chem Lett. 2022 Sep 27;13(39):8963–70. doi: 10.1021/acs.jpclett.2c01020 (PMC9549516; doi:10.1021/acs.jpclett.2c01020)
Supplement: Supplementary file 1 — jz2c01020_si_001.pdf [file jz2c01020_si_001.pdf]

Supplementary Information

Saturable Absorption of Free-Electron Laser Radiation by Graphite near the Carbon K-Edge

Lars Hoffmann[1,2,3], Sasawat Jamnuch[4], Craig P. Schwartz[2,5], Tobias Helk[6,7], Sumana L. Raj[1], Hikaru Mizuno[1,2], Riccardo Mincigrucci[8], Laura Foglia[8], Emiliano Principi[8], Richard J. Saykally[1,2], Walter S. Drisdell[2,9], Shervin Fatehi[10,‡], Tod A. Pascal[4,11,12,†], Michael Zuerch[1,2,3,13,*]

1. Department of Chemistry, University of California, Berkeley, California 94720, USA
2. Chemical Sciences Division, Lawrence Berkeley National Laboratory, Berkeley, California 94720, USA
3. Fritz Haber Institute of the Max Planck Society, 14195 Berlin, Germany
4. ATLAS Materials Science Laboratory, Department of Nano Engineering and Chemical Engineering, University of California, San Diego, La Jolla, California, 92023, USA
5. Nevada Extreme Conditions Laboratory, University of Nevada, Las Vegas, NV 89154, USA
6. Institute of Optics and Quantum Electronics, Abbe Center of Photonics, Friedrich-Schiller University, 07743 Jena, Germany
7. Helmholtz Institute Jena, 07743 Jena, Germany
8. Elettra-Sincrotrone Trieste S.C.p.A., Strada Statale 14, 34149 Trieste, Italy
9. Joint Center for Artificial Photosynthesis, Lawrence Berkeley National Laboratory, Berkeley, California 94720, USA
10. Department of Chemistry, The University of Texas Rio Grande Valley, Edinburg, Texas 78539, USA
11. Materials Science and Engineering, University of California San Diego, La Jolla, California, 92023, USA
12. Sustainable Power and Energy Center, University of California San Diego, La Jolla, California, 92023, USA
13. Materials Sciences Division, Lawrence Berkeley National Laboratory, Berkeley, California 94720, USA

Correspondence and requests for materials should be addressed to M. Zuerch (*mwz@berkeley.edu), Tod A. Pascal (†tpascal@ucsd.edu), and Shervin Fatehi (‡shervin.fatehi@utrgv.edu).

Computational Methods

We employed velocity-gauge real time time-dependent density functional theory (VG-RTTDDFT) within a numerical atomic orbital basis set to propagate the graphite under an intense laser field.[1–5] Exchange-correlation (XC) effects are treated at the level of the adiabatic local density approximation (ALDA) with the Perdew-Zunger LDA.[6] The carbon pseudopotential is explicitly generated from pseudoizing the C:{1s,2p,3d}, and the numerical basis sets are of double-ζ quality. The C 2s state is then obtained as a higher energy solution of the atomic Schrodinger equation.

The linear response of the system is extracted by propagating the graphite primitive cell under a small impulse function and then averaged over Cartesian fields, at which point the system is propagated for 25 fs under a $\sin^2$ envelope centered at t = 12.5 fs. An across-the-board energy shift of 20 eV is required to align the simulated result with the experiment. A 10 x 10 x 10 gamma point–centered k-point mesh was used to sample the Brillouin zone, with the mesh energy cut-off set to 400 Ry. We include a total of 120 empty bands extending to ~800 eV above the Fermi level to account for excited states. The incoming pulse intensities ranges from 10^{10}–10^{15} W/cm^2 at energies resonant with the simulated π* and σ* transitions. The theoretical intensity is determined from applied electric field strength of the sinosoidal envelope. The total energy of the envelope over time matches the intensity in the experiment and the one reported in Fig. 2c/2d.

Under ALDA, the energy inputted into the system is conserved, and we analyzed the time evolution of the absorbed energy per unit energy input, $E_{absorbed}(t) = E(t) – E(0)$, at three different energy pulses representing pre-edge, π*, and σ* features. To verify the result, we take the final energy of the system at t = 25 fs as representative of the system after X-ray

excitation. To match the total fluence and its spatial distribution to the experiment, the pulse envelope is designed to account for the cross-sectional area of the simulated unit cell.

We infer the transmitted intensity from the energy absorbed by the system and fit the transmission to Eq. (1) (reproduced below), which combines both saturable absorption and two-photon absorption effects[7,8]:

$$T(I) = \exp\left(-\left[\frac{\alpha_0}{1+I/I_{sat}} + \alpha_{NS} + \beta I\right] d\right)$$

Here *T(I)* is the transmission; α_0, α_{NS} and β are constants related to saturable, non-saturable, and two-photon absorption, respectively; and I_{sat} is a characteristic saturation intensity. (See **Table S1** for fitted values of these constants.) The thickness *d* is set to 0.68 nm, the thickness of graphite primitive cell used in the simulation.

We also investigate the effect of changing the polarization of the incident electric field on the energy absorption. In particular, we compare the energy absorbed by the simulated graphite sample from a short pulse (1 fs) at the π* excitation frequency with two different polarizations. The duration of 1 fs was chosen in order to limit the number of time steps required to observe a qualitative trend; we anticipate that slightly longer pulses (such as those used in the experiment) will exhibit similar behavior. As shown in **Fig. S4**, and consistent with our intuition, the energy absorption is more pronounced if the incoming field is polarized perpendicular to the graphite layer (i.e., along the c-axis), because the π* excitation is out-of-plane.[9] A pulse polarized parallel to the plane (i.e., in the ab plane) deposits approximately 7 times less energy into the graphite. Assessing the relative importance of additional decay channels discussed in the main text, such as Auger decay, will require further development of our computational methods.

Beam Diagnostics

At FERMI each individual FEL shot is characterized by the Photon Analysis Delivery and Reduction System (PADReS).[10] **Fig. S5** shows the shot-to-shot beam diagnostics for a representative dataset consisting of 443 FEL pulses. The FEL intensity was measured at two different positions. Typical intensity fluctuations are shown in **Fig. S5a**. The gas-based I0 monitor at PADReS and an ellipsoidal beamline mirror drain current at the EIS-TIMEX beamline were used to calibrate the incoming FEL intensity (**Fig. S5b**). The PRESTO spectrometer was used to analyze the beam profile, wavelength, and spectral bandwidth. The central wavelength jitter is $<10^{-4}$ nm, and the spectral bandwidth is close to $9\cdot10^{-4}$ nm (0.06 eV at a photon energy of 285.7 eV) (**Fig. S5c,d**). We analyzed the beam profile but did not filter shots that were not perfectly Gaussian in order to maintain a larger data set. In a previous experiment, it was found that two-photon absorption is significantly less sensitive to the beam mode than other nonlinear processes, such as second-harmonic absorption.[11,12] **Fig. S5** presents shot-to-shot beam diagnostics for one dataset consisting of 443 FEL pulses.

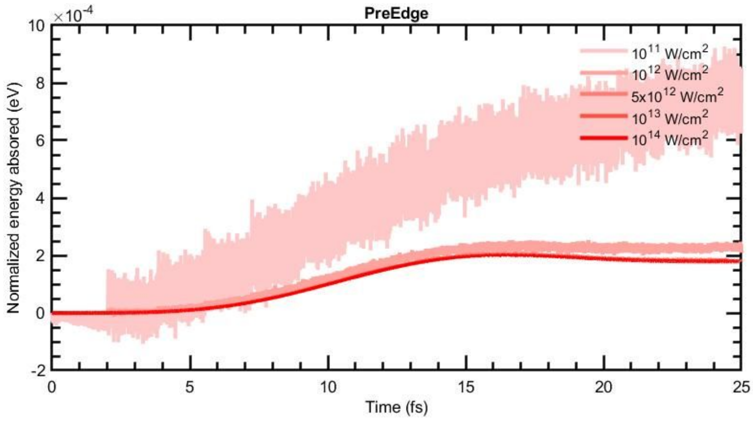

Figure S1: Calculated time evolution of energy absorbed at the pre-edge (261.6 eV). The absorption is low due to non-resonant conditions and appears largely independent of the pulse intensity. At lower intensities, the absorbed energy does not display a significant response, as the numerical noise within the simulation is on the same scale as the absorption.

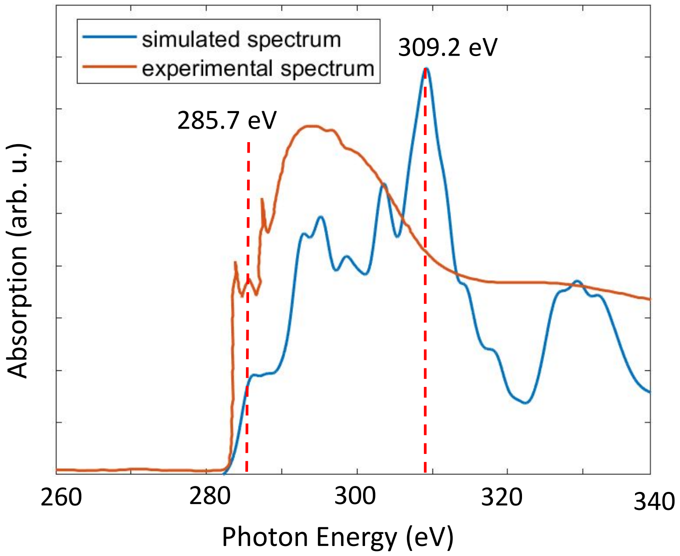

Figure S2: Experimental and simulated linear absorption spectrum of the graphite sample. An across-the-board shift of 20 eV is applied to the simulated spectrum. Red dotted lines indicate the photon energies used to stimulate π* (285.7 eV) and σ* (309.2 eV) transitions.

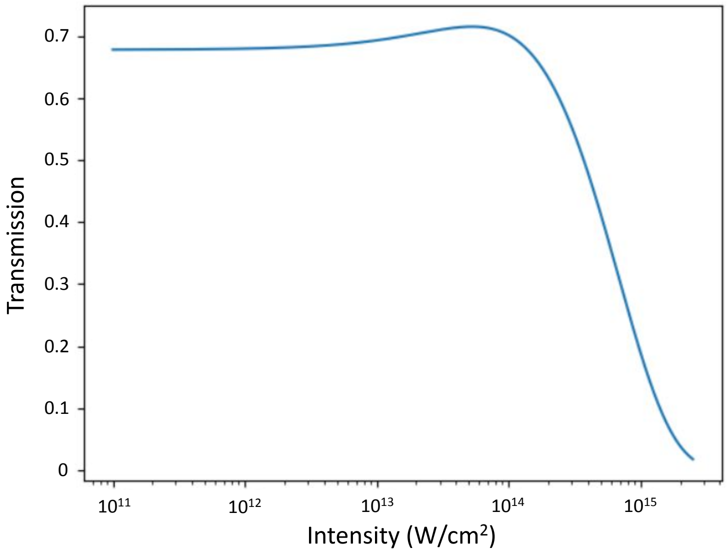

Figure S3: Calculated transmission for an 80 nm graphite foil obtained using **Eq. (1)** and the fit parameters from **Table S1**. The transmission is lower than that determined from simulations of the 0.68 nm graphite unit cell, but the overall lineshape does not differ from that shown in **Figs. 2c,d**.

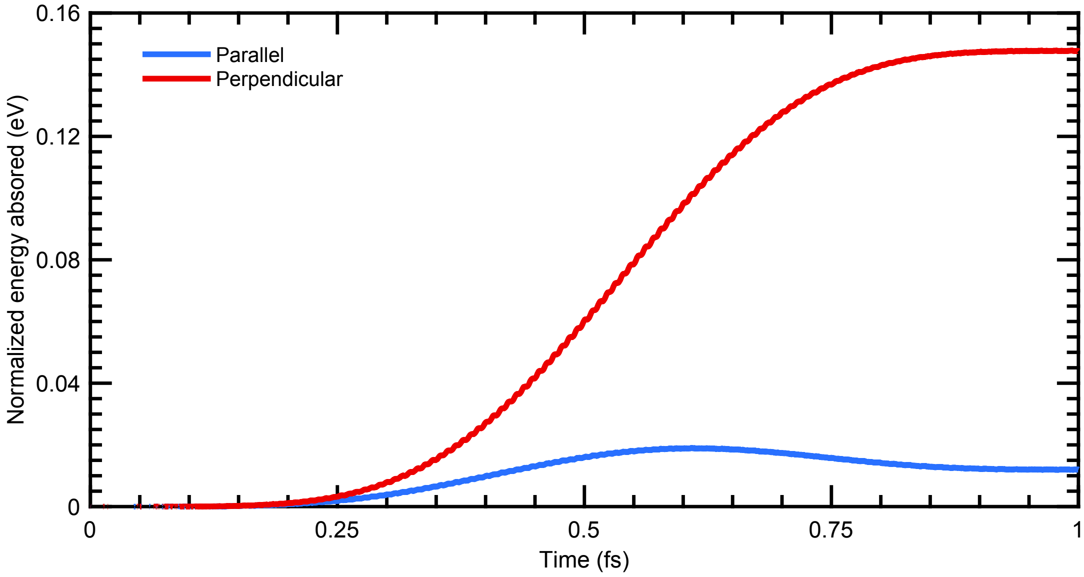

Figure S4: Energy absorption at the π* excitation with photons polarized parallel (blue) and perpendicular (red) to the graphite layer. Due to the out-of-plane character of the excitation, the energy absorption from photons with parallel polarization is reduced by a factor of ~7 relative to perpendicular polarization.

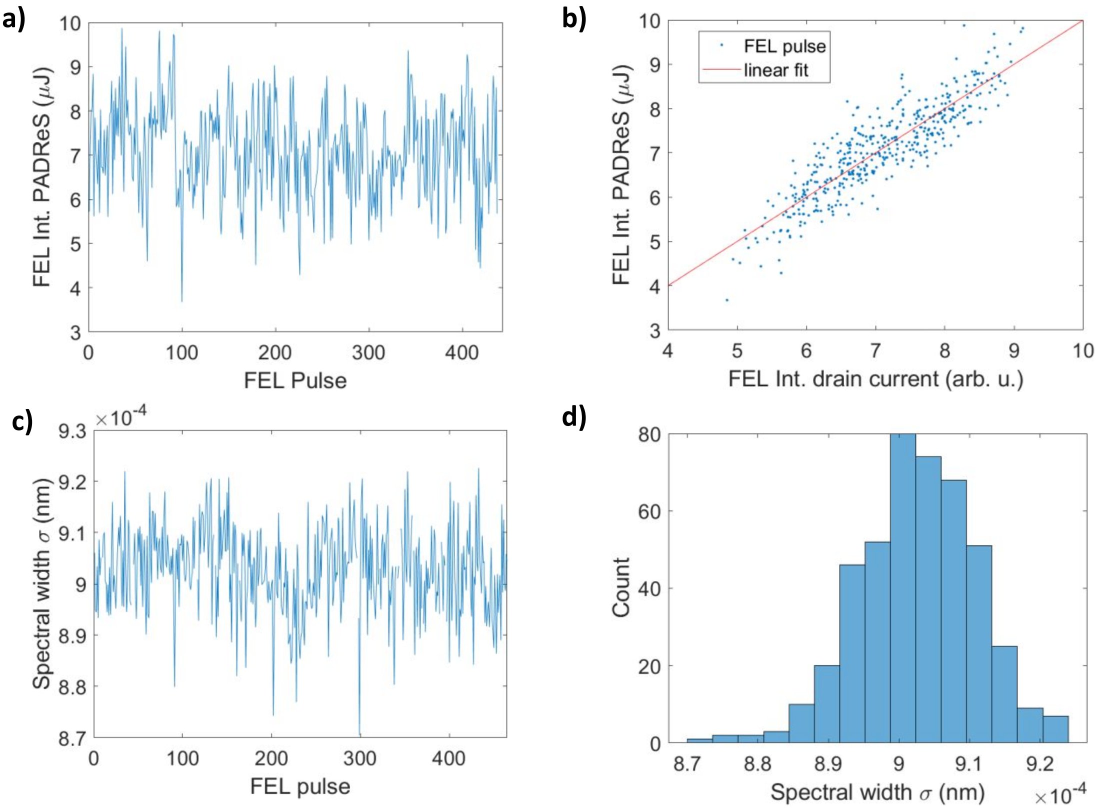

Figure S5: Shot-to-shot FEL beam diagnostics from PADReS. a) FEL pulse intensity variation for a typical set of FEL shots during the beamtime. b) FEL intensity calibration curve used for the analysis. c) Spectral bandwidth. d) Histogram of the spectral bandwidth as derived from PADReS beamline diagnostics.

Table S1: Model parameters used to fit transmission vs. intensity data from first-principles TD-DFT simulations.

	α_0, nm^{-1}	I_{sat} (W/cm^2)	α_{NS}, nm^{-1}	β $(W/cm^2)^{-1}$ nm^{-1}
C1s -> π*	$5.8219\cdot10^{-3}$	$5.70\cdot10^{15}$	$8.7143\cdot10^{-3}$	$4.3884\cdot10^{-7}$
C1s -> σ*	$3.033\cdot10^{-3}$	$1.56\cdot10^{16}$	$2.6708\cdot10^{-4}$	$1.3608\cdot10^{-6}$

References

(1) Yabana, K.; Bertsch, G. F. Time-Dependent Local-Density Approximation in Real Time. *Phys. Rev. B* **1996**, *54* (7), 4484–4487. https://doi.org/10.1103/PhysRevB.54.4484.

(2) Bertsch, G. F.; Iwata, J.-I.; Rubio, A.; Yabana, K. Real-Space, Real-Time Method for the Dielectric Function. *Phys. Rev. B* **2000**, *62* (12), 7998–8002. https://doi.org/10.1103/PhysRevB.62.7998.

(3) Soler, J. M.; Artacho, E.; Gale, J. D.; García, A.; Junquera, J.; Ordejón, P.; Sánchez-Portal, D. The SIESTA Method for *Ab Initio* Order- *N* Materials Simulation. *J. Phys. Condens. Matter* **2002**, *14* (11), 2745–2779. https://doi.org/10.1088/0953-8984/14/11/302.

(4) Pemmaraju, C. D.; Vila, F. D.; Kas, J. J.; Sato, S. A.; Rehr, J. J.; Yabana, K.; Prendergast, D. Velocity-Gauge Real-Time TDDFT within a Numerical Atomic Orbital

Basis Set. *Comput. Phys. Commun.* **2018**, *226*, 30–38. https://doi.org/10.1016/j.cpc.2018.01.013.
(5) Takimoto, Y.; Vila, F. D.; Rehr, J. J. Real-Time Time-Dependent Density Functional Theory Approach for Frequency-Dependent Nonlinear Optical Response in Photonic Molecules. *J. Chem. Phys.* **2007**, *127* (15), 154114. https://doi.org/10.1063/1.2790014.
(6) Perdew, J. P.; Zunger, A. Self-Interaction Correction to Density-Functional Approximations for Many-Electron Systems. *Phys. Rev. B* **1981**, *23* (10), 5048–5079. https://doi.org/10.1103/PhysRevB.23.5048.
(7) Boyd, R. W. *Nonlinear Optics*; Academic Press: New York, 2008.
(8) Malouf, A.; Henderson-Sapir, O.; Set, S.; Yamashita, S.; Ottaway, D. J. Two-Photon Absorption and Saturable Absorption of Mid-IR in Graphene. *Appl. Phys. Lett.* **2019**, *114* (9), 091111. https://doi.org/10.1063/1.5088641.
(9) Su, G. M.; Patel, S. N.; Pemmaraju, C. D.; Prendergast, D.; Chabinyc, M. L. First-Principles Predictions of Near-Edge X-Ray Absorption Fine Structure Spectra of Semiconducting Polymers. *J. Phys. Chem. C* **2017**, *121* (17), 9142–9152. https://doi.org/10.1021/acs.jpcc.7b01353.
(10) Zangrando, M.; Cocco, D.; Fava, C.; Gerusina, S.; Gobessi, R.; Mahne, N.; Mazzucco, E.; Raimondi, L.; Rumiz, L.; Svetina, C. Recent Results of PADReS, the Photon Analysis Delivery and REduction System, from the FERMI FEL Commissioning and User Operations. *J. Synchrotron Radiat.* **2015**, *22* (3), 565–570. https://doi.org/10.1107/S1600577515004580.
(11) Lam, R. K.; Raj, S. L.; Pascal, T. A.; Pemmaraju, C. D.; Foglia, L.; Simoncig, A.; Fabris, N.; Miotti, P.; Hull, C. J.; Rizzuto, A. M.; Smith, J. W.; Mincigrucci, R.; Masciovecchio, C.; Gessini, A.; De Ninno, G.; Diviacco, B.; Roussel, E.; Spampinati, S.; Penco, G.; Di Mitri, S.; Trovò, M.; Danailov, M. B.; Christensen, S. T.; Sokaras, D.; Weng, T.-C.; Coreno, M.; Poletto, L.; Drisdell, W. S.; Prendergast, D.; Giannessi, L.; Principi, E.; Nordlund, D.; Saykally, R. J.; Schwartz, C. P. Two-Photon Absorption of Soft X-Ray Free Electron Laser Radiation by Graphite near the Carbon K-Absorption Edge. *Chem. Phys. Lett.* **2018**, *703*, 112–116. https://doi.org/10.1016/j.cplett.2018.05.021.
(12) Lam, R. K.; Raj, S. L.; Pascal, T. A.; Pemmaraju, C. D.; Foglia, L.; Simoncig, A.; Fabris, N.; Miotti, P.; Hull, C. J.; Rizzuto, A. M.; Smith, J. W.; Mincigrucci, R.; Masciovecchio, C.; Gessini, A.; Allaria, E.; De Ninno, G.; Diviacco, B.; Roussel, E.; Spampinati, S.; Penco, G.; Di Mitri, S.; Trovò, M.; Danailov, M.; Christensen, S. T.; Sokaras, D.; Weng, T.-C.; Coreno, M.; Poletto, L.; Drisdell, W. S.; Prendergast, D.; Giannessi, L.; Principi, E.; Nordlund, D.; Saykally, R. J.; Schwartz, C. P. Soft X-Ray Second Harmonic Generation as an Interfacial Probe. *Phys. Rev. Lett.* **2018**, *120* (2), 023901. https://doi.org/10.1103/PhysRevLett.120.023901.
