# Supplementary material for: Saturable Absorption of Free-Electron Laser Radiation by Graphite near the Carbon K-Edge
Source: J Phys Chem Lett. 2022 Sep 27;13(39):8963–70. doi: 10.1021/acs.jpclett.2c01020 (PMC9549516; doi:10.1021/acs.jpclett.2c01020)
Supplement: Supplementary file 2 — jz2c01020_si_002.pdf [file jz2c01020_si_002.pdf]

jz-2022-010204.R1

Name: Peer Review Information for "Saturable Absorption of Free-Electron Laser Radiation by Graphite Near the Carbon K-Edge"

First Round of Reviewer Comments

Reviewer: 1

Comments to the Author

This paper presents the observation in the same experiment of both saturable absorption and 2-photon absorption. The data are clear and well presented and the theoretical rationalization is sound and convincing.

Reviewer: 2

Comments to the Author

In the present work, the transmission of x rays through an 80-nm-thick graphite film was investigated, at two distinct photon energies around the C 1s threshold, as a function of the x-ray intensity. The experimental results are shown in Fig. 2, panels (a) and (b). In contrast to what the y-axis labels suggest, what is called transmission in these two panels is not the same sort of quantity as what is called transmission in panels (c) and (d). In (a) and (b), "Transmission" is a measure of the number of transmitted photons, whereas in (c) and (d), which show computational results, "Transmission" is the ratio of the number of transmitted photons and the number of incident photons. I would also like to note that the mere fact that apparently no calibration of the transmitted fluence was performed (for whatever reason), is no justification for not using tick marks and tick labels for the vertical axes in panels (a) and (b).

The main insight provided by the experimental data is that at x-ray intensities of the order of 10^13 W/cm^2, the number of photons transmitted through the graphite film is no longer perfectly proportional to the number of incident photons: At the lower of the two photon energies investigated, which in the linear regime corresponds to excitation from 1s to pi^*-type orbitals, there is a recognizable suppression of transmission. In contrast, at the higher photon energy, which in the linear regime corresponds to excitation from 1s to sigma^*-type orbitals, the transmission is enhanced. So far so good. Using simulations, the authors attempt to come to a mechanistic understanding of these observations. But in spite of what the last two sentences of the paper, right before the acknowledgments, suggest, no detailed understanding has been achieved at all. I have several reasons for coming to this conclusion, as I will explain in the following.

(1) If we simply look at panels (c) and (d) in Fig. 2, all we see, in the intensity range investigated experimentally, is a slight enhancement of transmission, at both (!) photon energies. The relative

changes in transmission in panels (c) and (d) are of the order 10^{-3}. Yes, I realize that the calculations do not take into consideration the actual thickness of the graphite film used in experiment. But still, the suppression of transmission, seen in panel (a), is definitely not captured. And it is unclear whether the computed changes in transmission are not too small to account for the experimental observations. [I don't understand why the authors have not used Eq. (1), after having determined alpha_0 etc., to compute the transmission at the experimental value of the parameter d.] Based on the calculations performed, it is impossible to claim that a mechanistic understanding of the experimental data has been achieved.

(2) There are good reasons to believe that the calculations the authors have performed are, in fact, unable to describe the physics underlying the experiment. (More on this below.) However, if one does take them seriously, then, if we look at Fig. 3(c), we are forced to conclude that even at 10^14 W/cm^2, the response at 2*omega is suppressed, relative to the 1*omega peak, by close to five orders of magnitude. In fact, the relative contribution of the 2*omega response is more suppressed at the pi^* transition than at the sigma^* transition. Apart from the fact that 10^14 W/cm^2 is much higher than what was investigated experimentally, one would not expect to see much of a 2*omega contribution at the pi^* transition, but maybe at the sigma^* transition. This is, of course, precisely the opposite of the conclusion that the authors would like to draw based on their experimental data.

(3) The main reason I am saying that the calculations performed are most likely unable to explain the experiment is the extensive experience already gained in the field of nonlinear x-ray physics, primarily using gas-phase experiments and associated theory (which the authors may not be aware of). The basic physics that, in a dense sample leads to enhancement or suppression of transmission, can be characterized by measuring ion charge-state distributions and electron spectra [e.g., Nature 466, 56 (2010); Phys. Rev. Lett. 106, 083002 (2011)]. Those studies were carried out at higher intensities than those considered here. But since a fairly quantitative mechanistic understanding was demonstrated at those higher intensities, there is no particular reason to expect that what has been learned is completely inapplicable at lower intensities. One of the main insights gained was that the sort of single-electron two-photon process illustrated in Fig. 1(c) is very unlikely to take place. After an electron has been excited to unoccupied valence states, the probability that such an electron will absorb a second photon is relatively small. If xray pumping causes the K-shell excitation channel to close, then it is more likely, by at least a factor of 4, that the second photon would be absorbed by a sigma or a pi electron, rather than the pi^* or sigma^* electron. In other words, x-ray multiphoton physics is many-electron excitation physics. And many-electron excitation physics cannot be correctly captured by the TDDFT modeling employed by the authors. I agree that TDDFT would give the picture shown in Fig. 1(c). But that doesn't mean that that picture makes sense.

(4) Another reason why the calculations performed should not be expected to provide mechanistic insight is that the experimental x-ray pulse duration was longer, by a factor of three or so, than the C 1s Auger lifetime. This means that it is likely that after a 1s hole is formed, Auger decay takes place before (!) another photon is absorbed. The gas-phase studies alluded to above demonstrated that x-ray multiphoton physics is strongly affected by the competition between photoabsorption and Auger decay. Only when the x-ray pulse is shorter (ideally, much shorter) than the Auger lifetime does it make sense to neglect Auger decay (as far as x-ray absorption/transmission is concerned). Auger decay is a consequence of strong electron-electron interaction in core-excited states that are degenerate with

continuum states, converting one-hole configurations to two-hole one-particle configurations. That sort of physics is not captured by the TDDFT calculations the authors have performed.

(5) The question is: What is more important? Photoabsorption from sigma or pi orbitals (after Kshell photoabsorption), or Auger decay? In order to answer that question, one must understand what Auger decay would do to x-ray absorption. For every K hole, Auger decay gives rise to two holes almost anywhere in the valence band (not just holes at the top of the valence band, which may be thermally excited). Such holes lead to distinct changes in the x-ray absorption spectrum. On the one hand, one would expect the K edge to experience a blueshift in energy (which can lead to channel closing and thus a suppression of absorption). On the other hand, one would expect to see the formation of additional K-shell excitation channels, corresponding to the excitation of a 1s electron into one of the two holes. Experience from gas-phase studies shows that the K-shell absorption cross section for resonant excitation into valence holes can be much higher than the ground-state x-ray absorption cross would lead one to expect. To this end, I suggest that the authors take a look at Fig. 2(c) in Phys. Rev. Lett. 107, 233001 (2011): Strong new resonances are unveiled by poking holes into the valence shell. Whether one creates holes in the valence shell through valence photoabsorption (as was done in that PRL) or via Auger decay is not essential. What is essential is that such valence holes can lead to enhanced K-shell photoabsorption, depending, of course, on the x-ray energy used.

(6) Finally, it is quite unclear why it would make sense to use the experimental photon energies in the calculations, as stated on p. 3. This would be plausible only if, in the calculations, the experimental energies corresponded to the same classes of transitions. But the authors have not provided any evidence for that. In Fig. 1(b), we are shown an experimental x-ray absorption spectrum. Hence, in order to allow readers to judge whether the present TDDFT calculations can reproduce at least the linear absorption spectrum, without any need for an energy shift, they would have to compute the linear absorption spectrum and show it. If the authors applied a manual energy shift, they must say so. If they did not, then it is unlikely that the transitions considered in the calculations have much to do with the transitions investigated experimentally. Moreover, it is not enough to talk about the compact basis functions employed. The reader needs to be told how delocalized states were described. If a plane-wave basis was used, how dense was the k-grid within the first Brillouin zone? How many bands were included? Was an energy cutoff introduced in the orbital space used for TDDFT? Are the results converged with respect to the choices made?

Reviewer: 3

Comments to the Author

The manuscript describes the results of a transmission-geometry absorption experiment on graphite samples at the Carbon K-edge at a range of incident pulse intensities. These measurements are performed at two incident photon wavelengths and the observed trends in the changes in transmission are ascribed to relative competition between saturable absorption and two-photon absorption, supported by the results of velocity-gauge real time time-dependent density functional theory calculations. Overall, it is a clear, concise manuscript with compelling experimental results.

What is the major advance reported in the paper?

This is the first study that discusses the impact of saturable absorption at the carbon K edge on graphite samples at the two FEL wavelengths used. These are challenging experiments, and the experimental data is relatively high quality and there are clear differences in the spectra as a function of the FEL intensity.

What is the immediate significance of this advance?

Saturable absorption is of wide interest in terms of understanding fundamental non-linear phenomena in materials but is also important from the perspective of its application in many materials for optics. It has only recently has it been possible to generate sufficiently intense pulses at these photon wavelengths to investigate such phenomena experimentally, in particular the relationship between saturable absorption and two photon absorption processes. This, and the development of new theoretical frameworks for the calculation of and insight to these nonlinear processes, are important advances for nonlinear optics.

Technical suggestions

There is no discussion in the text about whether any of the changes in the relative behaviour of the 2PA versus SA could be as a result of anything other than the relative cross sections of the two transitions selected in the experiment. While these are both assigned as being bound transitions, the 1s->pi* is below the ionization threshold and the other is above? Is this potentially relevant at all to these processes and how generalisable do the authors think their results from this work (allowing for some differences in the core hole lifetime) are if this is truly just cross section dependent?

Are there any characterisations of the X-ray pulses at all? In particular their intensity distribution with time? Are the simulations insensitive to different structure in the envelope of the pulse?

It would be helpful in Fig 1c) to clarify the meaning of the blue box on the diagram – is this supposed to be the ionization threshold?

It would be good to emphasise more clearly some of the particular advances in this study over previous measurements by the authors and whether any reconsideration of this previous data should be in light of the new findings (https://doi.org/10.1016/j.cplett.2018.05.021)

The readability of the figure labels and text could be improved by using a larger font. Figures also seem to be quite low resolution?

Author's Response to Peer Review Comments:

Author reply for manuscript jz-2022-010204 by Hoffmann et al. “Saturable Absorption of Free-Electron Laser Radiation by Graphite Near the Carbon K-Edge”

Here we reply to the reviewers. We believe we have adequately addressed their comments as regards to our manuscript and have taken advantage of the opportunity to improve the quality of our arguments and the text. We have reproduced each of the reviewers’ comments in black italics and provide our detailed replies in blue for clarity, along with the details of any responsive changes made to the manuscript. At the end of this author's response, we also list changes made to accommodate editorial requests. Other minor changes, principally improvements to the wording, have also been made to the manuscript. All significant changes in the manuscript and SI are marked with a yellow background and have been reproduced here for consistency.

We wish to thank the referees for providing valuable feedback.

Reviewer 1

This paper presents the observation in the same experiment of both saturable absorption and 2-photon absorption. The data are clear and well presented and the theoretical rationalization is sound and convincing.

We thank the reviewer for the time spent reading our manuscript and for providing positive feedback.

Reviewer 2

In the present work, the transmission of x rays through an 80-nm-thick graphite film was investigated, at two distinct photon energies around the C 1s threshold, as a function of the x-ray intensity. The experimental results are shown in Fig. 2, panels (a) and (b). In contrast to what the y-axis labels suggest, what is called transmission in these two panels is not the same sort of quantity as what is called transmission in panels (c) and (d). In (a) and (b), "Transmission" is a measure of the number of transmitted photons, whereas in (c) and (d), which show computational results, "Transmission" is the ratio of the number of transmitted photons and the number of incident photons. I would also like to note that the mere fact that apparently no calibration of the transmitted fluence was performed (for whatever reason), is no justification for not using tick marks and tick labels for the vertical axes in panels (a) and (b).

We thank the reviewer for catching this inconsistency, which we have corrected in the revised manuscript. We changed the y-axis labels in Fig. 2 panels a) and b) to make the distinction between the two quantities more clear. We have also added tick marks in panels a) and b) as suggested.

In panels c) and d), we report the percent transmission of the number of photons after excitation for the duration of the pulse. Unfortunately, we cannot plot the experimental transmission in the

same way as in panel c) and d), due to the lack of calibration allowing us to determine the absolute number of photons downstream of the sample. Such absolute calibration is not available at the infrastructure used for the experiments and in general, absolute calibration of photon detectors is rarely available at FEL facilities, to the best of our knowledge. Therefore, we plot the transmitted vs. incoming intensity (uncalibrated) obtained by the spectrometer CCD and I_0 monitor, respectively, in order to observe a trend between the two quantities. Consequently, a linear trend in the panel a) and b) translates to a constant trend in panel c) and d). This distinction should not limit our conclusions. We added the following sentences to the discussion sections of the manuscript to clarify this:

*"We point out that **Figs. 2a,b** plot the transmitted X-ray intensity while **Figs. 2c,d** plot the fraction of transmitted and incoming intensity. Consequently a linear trend in **Figs 2a,b** corresponds to a constant trend in **Figs 2c,d**."*

The main insight provided by the experimental data is that at x-ray intensities of the order of 10^13 W/cm^2, the number of photons transmitted through the graphite film is no longer perfectly proportional to the number of incident photons: At the lower of the two photon energies investigated, which in the linear regime corresponds to excitation from 1s to pi^*-type orbitals, there is a recognizable suppression of transmission. In contrast, at the higher photon energy, which in the linear regime corresponds to excitation from 1s to sigma^*-type orbitals, the transmission is enhanced. So far so good. Using simulations, the authors attempt to come to a mechanistic understanding of these observations. But in spite of what the last two sentences of the paper, right before the acknowledgments, suggest, no detailed understanding has been achieved at all. I have several reasons for coming to this conclusion, as I will explain in the following.

We thank the reviewer for this frank assessment. We do, however, respectfully disagree with the judgment that no detailed understanding has been achieved at all. As the reviewer notes, we have experimentally observed an increase in transmission for the 1s to σ* excitation and a decrease of transmission for the 1s to π* excitation. By combining our real-time TDDFT simulations with a transmission model accounting for saturable and two-photon absorption effects, we found that increasing the intensity initially leads to an increase in transmission attributable to saturable absorption, while at even higher intensities the transmission decreases as two-photon absorption begins to dominate. Comparing simulations for the two transitions shows that TPA is significant at lower intensities for the σ* transition than for the π*. While we do not observe the decrease in transmission associated with TPA in the experimental data for σ*, we have provided several possible explanations of why this could be, in particular that the series of pulses delivered by the FEL at the σ* excitation frequency were of somewhat lower intensity than for the π*. We have revised the manuscript in various places to render our argument more clearly and concisely.

(1) If we simply look at panels (c) and (d) in Fig. 2, all we see, in the intensity range investigated experimentally, is a slight enhancement of transmission, at both (!) photon energies. The relative changes in transmission in panels (c) and (d) are of the order 10^{-3}. Yes, I realize that the

calculations do not take into consideration the actual thickness of the graphite film used in experiment. But still, the suppression of transmission, seen in panel (a), is definitely not captured. And it is unclear whether the computed changes in transmission are not too small to account for the experimental observations. [I don't understand why the authors have not used Eq. (1), after having determined alpha_0 etc., to compute the transmission at the experimental value of the parameter d.] Based on the calculations performed, it is impossible to claim that a mechanistic understanding of the experimental data has been achieved.

We thank the reviewer for this comment, which raises an issue we agree was not clearly discussed in the original manuscript. To address the first comment relating to the intensity, we emphasize that due to instrumentation constraints, we were not able to explore a wider range of FEL intensities. In the range of intensities measured experimentally, our calculations show an initial increase in the transmission, followed by a more pronounced increase in transmission for the σ^* and a significant decrease in transmission for the π^*, both of which qualitatively capture the experiments. As noted above, several factors may account for any detailed discrepancy between simulations and experiments, but we are confident that the trends are consistent.

To address the second comment relating to the use of Eq. (1), we have taken the reviewer's wise suggestion and used the fitted parameters from the simulation to calculate the transmission at the experimental value of the sample thickness. Because the thickness *d* is an overall multiplicative constant in the exponential in Eq. (1), only the magnitude of the resulting transmission changes, not the lineshape, as shown in the figure below. We have added the following sentence to the description of the fitting model to emphasize this point:

"The thickness of the calculated graphite model reduces the total transmission relative to that of the experimental sample (80 nm) but does not change the lineshape."

We have also added the following graph (**Fig. S3**) to the Supporting Information:

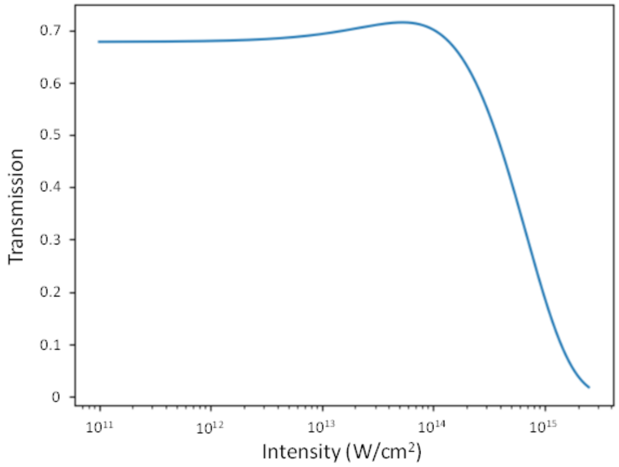

Figure S3: *Calculated transmission for an 80 nm graphite foil obtained using* ***Eq. (1)*** *and the fit parameters from* ***Table S1****. The transmission is lower than that determined from simulations of*

*the 0.68 nm graphite unit cell, but the overall lineshape does not differ from that shown in **Figs. 2c,d**.*

*(2) There are good reasons to believe that the calculations the authors have performed are, in fact, unable to describe the physics underlying the experiment. (More on this below.) However, if one does take them seriously, then, if we look at Fig. 3(c), we are forced to conclude that even at 10^14 W/cm^2, the response at 2*omega is suppressed, relative to the 1*omega peak, by close to five orders of magnitude. In fact, the relative contribution of the 2*omega response is more suppressed at the pi^* transition than at the sigma^* transition. Apart from the fact that 10^14 W/cm^2 is much higher than what was investigated experimentally, one would not expect to see much of a 2*omega contribution at the pi^* transition, but maybe at the sigma^* transition. This is, of course, precisely the opposite of the conclusion that the authors would like to draw based on their experimental data.*

We thank the reviewer for their comment. We respectfully disagree with the assessment that the numerical model is unable to describe the physics correctly. The reviewer has additional comments to that effect in the following points, where we reply in more detail regarding the model and its capabilities. Here, we would like to respond to concerns regarding **Fig. 3**. Indeed, there was a mistake in the original figure, where we accidentally inserted a non-normalized panel. We have corrected this oversight. In particular, we replaced the plots in 3c) and 3d) with curves normalized to their maximum peak amplitude and clarified the observation that in general the TPA process is more prevalent at σ* excitation. It is also worth pointing out that in the simulation, we have assumed an angle of incidence such that the electric field of the FEL maximizes the absorbance for both π* and σ* transition; we now discuss this issue in detail in the SI and have done *additional simulations* to corroborate our finding (**Fig. S4**).

In **Figs. 3c,d** we chose for clarity to present the Fourier transforms at an intensity where the $\omega$and 2ω signals are nicely visible (10^{14} W/cm²). In **Fig. 2**, each the data point is extracted from a similar calculation, and we can see clear trends. Therefore, we do not find it limiting to show a single representative example for the frequency domain in **Figs. 3c,d**.

In addition, the comment by the reviewer motivated us to revisit another possible source of error: In the experiment a polycrystalline sample was used, while our simulations considered a perfect crystalline structure with the polarization oriented to maximize the absorption for each transition. We acknowledge that there could thus be a slight overestimate of the absorption relative to the experiment. We have added the following discussion of this point to the manuscript:

"Another possible source of inconsistencies between the experiment and simulations of the TPA process is the varying thickness and structuring of the sample. In the simulation, we assume a crystalline graphite sample oriented such that the polarization of the electric field of the FEL is perpendicular to the graphite layer (i.e. along the c-axis) for the π excitation and parallel to it (i.e. in the ab plane) for the σ* excitation. This geometry maximizes the overall absorption, which may lead to an overestimation of absorption-induced effects from theory.[32] In the experiment, by contrast, the sample is polycrystalline and by definition includes some randomness in its structure.*

To gauge the significance of this difference, we simulate the effect of changing the polarization of the incoming photon on the energy absorbed. We observe that the absorption is indeed greatly reduced if the photon's field of polarization is not well-aligned with the respective excitation in the sample. The simulated linear absorption spectrum, additional details of the numerical simulation parameters, and comparison of absorbed energies as a function of incident photon polarization can be found in the Supporting Information."

We have also added the following simulation detail to the SI:

"We also investigate the effect of changing the polarization of the incident electric field on the energy absorption. In particular, we compare the energy absorbed by the simulated graphite sample from a short pulse (1 fs) at the π excitation frequency with two different polarizations. The duration of 1 fs was chosen in order to limit the number of time steps required to observe a qualitative trend; we anticipate that slightly longer pulses (such as those used in the experiment) will exhibit similar behavior. As shown in **Fig. S4**, and consistent with our intuition, the energy absorption is more pronounced if the incoming field is polarized perpendicular to the graphite layer (i.e., along the c-axis), because the π* excitation is out-of-plane.[9] A pulse polarized parallel to the plane (i.e., in the ab plane) deposits approximately 7 times less energy into the graphite."*

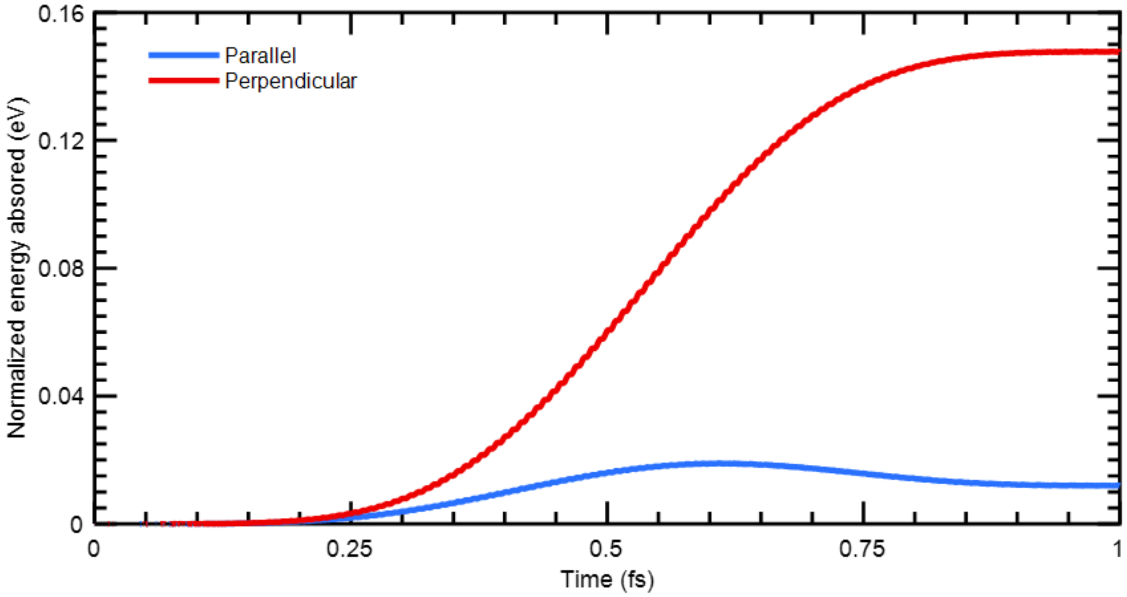

Figure S4: *Energy absorption at the π* excitation with photons polarized parallel (blue) and perpendicular (red) to the graphite layer. Due to the out-of-plane character of the excitation, the energy absorption from photons with parallel polarization is reduced by a factor of ~7 relative to perpendicular polarization.*

(3) The main reason I am saying that the calculations performed are most likely unable to explain the experiment is the extensive experience already gained in the field of nonlinear x-ray physics, primarily using gas-phase experiments and associated theory (which the authors may not be

aware of). The basic physics that, in a dense sample leads to enhancement or suppression of transmission, can be characterized by measuring ion charge-state distributions and electron spectra [e.g., Nature 466, 56 (2010); Phys. Rev. Lett. 106, 083002 (2011)]. Those studies were carried out at higher intensities than those considered here. But since a fairly quantitative mechanistic understanding was demonstrated at those higher intensities, there is no particular reason to expect that what has been learned is completely inapplicable at lower intensities. One of the main insights gained was that the sort of single-electron two-photon process illustrated in Fig. 1(c) is very unlikely to take place. After an electron has been excited to unoccupied valence states, the probability that such an electron will absorb a second photon is relatively small. If xray pumping causes the K-shell excitation channel to close, then it is more likely, by at least a factor of 4, that the second photon would be absorbed by a sigma or a pi electron, rather than the pi^* or sigma^* electron. In other words, x-ray multiphoton physics is many-electron excitation physics. And many-electron excitation physics cannot be correctly captured by the TDDFT modeling employed by the authors. I agree that TDDFT would give the picture shown in Fig. 1(c). But that doesn't mean that that picture makes sense.

The reviewer is correct that the vg-RT-TDDFT approach employed in this work does not allow for any “shake-up” or “shake-down” processes associated with secondary events, such as Auger decay, valence hole excitations, or stimulated emission. The reviewer also raises the interesting possibility of additional absorption processes leading to an increase in absorption at the π^*, namely, excitations from valence holes spontaneously formed from Auger decay events. We believe, however, that Auger processes do not play a significant role in the observed experimental nonlinearities, for several reasons: First, they would not explain the decrease in absorption observed at the π^* resonance. Second, our X-ray beam was tuned into resonance with the π^* and σ^* excitations, and the seeded FERMI FEL used for the study has very small photon energy fluctuations relative to FELs of the SASE type. Thus, excitations into non-resonant levels are expected to have a very small cross section. Third, there is a possibility that the filled core resulting from Auger decay would be subsequently excited into unoccupied orbitals by another incoming photon. But while we cannot discount this possibility, we note that such a linear adsorption event cannot explain the nonlinear response observed experimentally.

Thus, given the scope of the calculations, which we emphasize reproduces the trends shown in the experiments, we are confident that the proposed competition between SA and TPA is the determining physics in this system. We do appreciate the point raised by the reviewer, though, and we believe it useful to mention it in the manuscript for the benefit of the *JPCL* readership. We therefore added the following sentence along with the suggested citations:

“Auger decay events—and any sequential absorption processes following the formation of the resulting valence hole[28,29]—are not captured by the level of theory applied here. That said, Auger decay is unlikely to have played a significant role in these experiments due to the narrow spectral width and high spectral stability of the FEL source tuned into resonance with the 1s to π^* and 1s to σ^* transitions. ”

(4) Another reason why the calculations performed should not be expected to provide mechanistic insight is that the experimental x-ray pulse duration was longer, by a factor of three or so, than the C 1s Auger lifetime. This means that it is likely that after a 1s hole is formed, Auger decay takes place before (!) another photon is absorbed. The gas-phase studies alluded to above demonstrated that x-ray multiphoton physics is strongly affected by the competition between photoabsorption and Auger decay. Only when the x-ray pulse is shorter (ideally, much shorter) than the Auger lifetime does it make sense to neglect Auger decay (as far as x-ray absorption/transmission is concerned). Auger decay is a consequence of strong electron-electron interaction in core-excited states that are degenerate with continuum states, converting one-hole configurations to two-hole one-particle configurations. That sort of physics is not captured by the TDDFT calculations the authors have performed.

As noted in the response above, we do not agree that Auger decay and subsequent linear absorption events are consistent with the nonlinear increase/decrease in transmission observed for the π^* and σ^* resonances in our experiments.

(5) The question is: What is more important? Photoabsorption from sigma or pi orbitals (after Kshell photoabsorption), or Auger decay? In order to answer that question, one must understand what Auger decay would do to x-ray absorption. For every K hole, Auger decay gives rise to two holes almost anywhere in the valence band (not just holes at the top of the valence band, which may be thermally excited). Such holes lead to distinct changes in the x-ray absorption spectrum. On the one hand, one would expect the K edge to experience a blueshift in energy (which can lead to channel closing and thus a suppression of absorption). On the other hand, one would expect to see the formation of additional K-shell excitation channels, corresponding to the excitation of a 1s electron into one of the two holes. Experience from gas-phase studies shows that the K-shell absorption cross section for resonant excitation into valence holes can be much higher than the ground-state x-ray absorption cross would lead one to expect. To this end, I suggest that the authors take a look at Fig. 2(c) in Phys. Rev. Lett. 107, 233001 (2011): Strong new resonances are unveiled by poking holes into the valence shell. Whether one creates holes in the valence shell through valence photoabsorption (as was done in that PRL) or via Auger decay is not essential. What is essential is that such valence holes can lead to enhanced K-shell photoabsorption, depending, of course, on the x-ray energy used.

We thank the reviewer for this very helpful discussion. We refer to the responses above in regard to our interpretation of the interplay of the various processes. Additional shifts could, for example, stem from core-hole screening in the photoexcited state, or from lifetime broadening of the resonances. The full range of possible phenomena, especially those involving many-body interactions, are to our knowledge not captured by any existing theory to the extent that one could correctly predict all impacts. In the present work, which employs state-of-the-art numerical methods, one point we can make is that the photon energies chosen for the FEL experiments coincide with the resonance peaks observed in linear XAS spectra computed for the same sample (see new **Fig. S2**), which we align with the TDDFT-based calculation to capture the correct responses. Any such across-the-board shifts between experiment and simulation should be consistent, and we therefore anticipate being in resonance also in the photoexcited case.

Moreover, screening effects would be expected to impact absorption spectra on sub-femtosecond timescales, meaning that energy shifts from that origin should be similar between the slow synchrotron XAS measurement and the ~25fs FEL measurement. For Auger decay channels one would expect different behavior, as in the FEL case the measurement occurs within a few decay constants of the excited state. However, if in this case energies were indeed shifted off-resonance, one would not expect to observe any TPA at all. We have added discussion of this point to the manuscript, detailing these effects:

"A simulated linear XAS spectrum for the same type of sample exhibits the 1s to π and 1s to σ* transitions (see Supplementary Information* ***Fig. S2****) for which the FEL experiments were performed. Therefore, any energy-level shifts arising from core-hole effects or secondary excitations such as Auger decay should be captured at least qualitatively correctly. Some uncertainty remains due to the femtosecond nature of the photoexcitation pulse, which may entail a slightly different balance of secondary excitation events than in standard XAS; sub-femtosecond screening effects, however, should be largely independent of the time scales present in the experiment.[30,31]"*

(6) Finally, it is quite unclear why it would make sense to use the experimental photon energies in the calculations, as stated on p. 3. This would be plausible only if, in the calculations, the experimental energies corresponded to the same classes of transitions. But the authors have not provided any evidence for that. In Fig. 1(b), we are shown an experimental x-ray absorption spectrum. Hence, in order to allow readers to judge whether the present TDDFT calculations can reproduce at least the linear absorption spectrum, without any need for an energy shift, they would have to compute the linear absorption spectrum and show it. If the authors applied a manual energy shift, they must say so. If they did not, then it is unlikely that the transitions considered in the calculations have much to do with the transitions investigated experimentally. Moreover, it is not enough to talk about the compact basis functions employed. The reader needs to be told how delocalized states were described. If a plane-wave basis was used, how dense was the k-grid within the first Brillouin zone? How many bands were included? Was an energy cutoff introduced in the orbital space used for TDDFT? Are the results converged with respect to the choices made?

We appreciate this thoughtful comment. We expanded our discussion and have now included the simulated linear absorption in the SI as below (**Fig. S2**). As the reviewer correctly indicates, we had to correct a zero-energy shift typical for DFT-based methods. In more detail, we applied an across-the-board 20 eV shift to the spectra to align the simulated response with the experiment. A 10 x 10 x 10 gamma point–centered k-point mesh was used to sample the Brillouin zone, with the mesh energy cutoff set to 400 Ry. We include a total of 120 empty bands extending to ~800 eV above the Fermi level to account for excited states. We added these additional details verbatim to the SI.

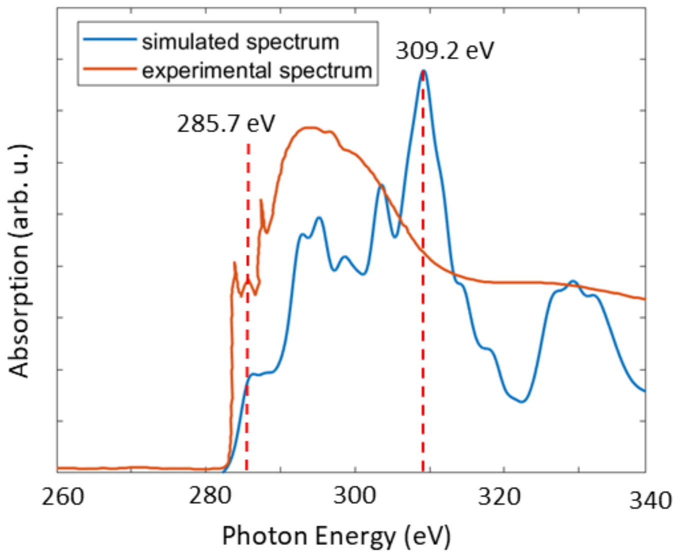

Figure S2: *Experimental and simulated linear absorption spectrum of the graphite sample. An across-the-board shift of 20 eV is applied to the simulated spectrum. Red dotted lines indicate the photon energies used to stimulate π* (285.7 eV) and σ* (309.2 eV) transitions.*

Reviewer 3

The manuscript describes the results of a transmission-geometry absorption experiment on graphite samples at the Carbon K-edge at a range of incident pulse intensities. These measurements are performed at two incident photon wavelengths and the observed trends in the changes in transmission are ascribed to relative competition between saturable absorption and two-photon absorption, supported by the results of velocity-gauge real time time-dependent density functional theory calculations. Overall, it is a clear, concise manuscript with compelling experimental results.

We thank the reviewer for their very positive response and for their time spent reading the manuscript. We respond to the technical comments below.

Technical suggestions:

There is no discussion in the text about whether any of the changes in the relative behaviour of the 2PA versus SA could be as a result of anything other than the relative cross sections of the two transitions selected in the experiment. While these are both assigned as being bound transitions, the 1s->pi is below the ionization threshold and the other is above? Is this potentially relevant at all to these processes and how generalisable do the authors think their results from this work (allowing for some differences in the core hole lifetime) are if this is truly just cross section dependent?*

The reviewer makes a very interesting point. Indeed, Reviewer 2 pointed us in a similar direction and prompted discussion of other absorption channels that become available subsequent to Auger decay. We also realized we neglected to discus core-hole screening effects that may change the energies of the resonances. We have performed additional calculations and added discussion of these additional considerations to the manuscript, and we kindly refer Reviewer 3 to responses #2, #3, and #5 above to Reviewer 2. To summarize, there are no exsting numerical approaches that can capture the full complexity of decay channels and many-body interactions on the X-ray absorption spectrum, especially once lifetime effects come into play. Our work focuses on understanding those nonlinear effects that arise from the response of the matter on short time scales, for which TDDFT is the state-of-the-art method. (It does, however, capture fewer of the many body interactions than other implementations of steady-state DFT.) We are confident in our experimental observations and are also positive that our simulations reproduce the observed trends correctly and for the right reasons. Regarding possible contributions of additional decay channels and other many-body effects, we can only make qualitative arguments at this point; developing more expansive theoretical methods is beyond the scope of the present work.

Are there any characterisations of the X-ray pulses at all? In particular their intensity distribution with time? Are the simulations insensitive to different structure in the envelope of the pulse?

We thank the reviewer for this thoughtful comment. Characterizing femtosecond FEL pulses in the XUV/X-ray domain is a significant challenge on its own, and such an effort is not typically part of the very limited beamtime allowed to users of FEL facilities. That said, the beamline scientists regularly conduct measurements to confirm the reliable operation of their systems, and we defer to their previously published results, which we have added to our references (De Ninno, G., et al. Single-shot spectro-temporal characterization of XUV pulses from a seeded free-electron laser. Nat Commun 6, 8075 (2015). https://doi.org/10.1038/ncomms9075). It certainly is important to capture the pulse envelope, as its shape determines the pulse intensity, which in turn is essential for quantifying nonlinear processes. What makes the FERMI FEL special is that it is seeded by a laser source, giving it very clean pulses both spectrally and temporally. As such, we can assume a Gaussian envelope for the experiment (as discussed in the above-linked reference), which is very similar to the $\sin^2$ envelope is used in the simulations. It is worth noting that most other X-ray FELs operate in the self-amplified spontaneous emission regime, where typically the spectral and time characteristics of the pulse exhibit much more randomness, as the pulses are self-seeded from noise. That distinction between the FERMI FEL and competing X-ray FELs was key for our ability to perform the present research. To improve the manuscript, we have added the pulse characterization reference and a brief explanatory sentence:

"Knowledge of the pulse envelope is essential for correctly determining the pulse intensity; the seeded FERMI FEL is known to deliver pulses with a Gaussian envelope, similar to the $\sin^2$ envelopes commonly used in theoretical calculations.[20]"

The following figure shows the intensity distribution of the FEL with time for a dataset consisting of ~400 shots.

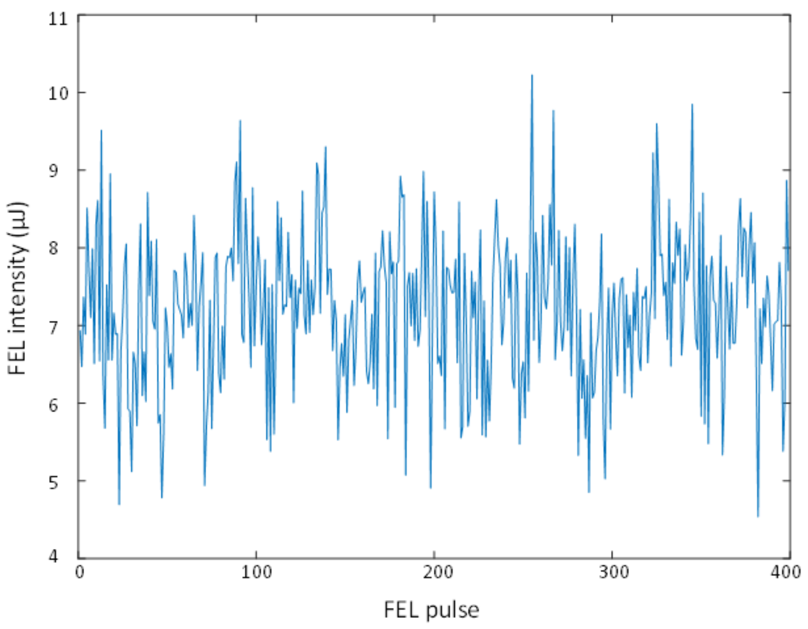

It would be helpful in Fig 1c) to clarify the meaning of the blue box on the diagram – is this supposed to be the ionization threshold?

Assuming the reviewer is referring to the dark blue box partially obscured by the label reading "285.7 eV," it is merely indicating the overlap of the blue bands for representing the *π* and *π** around the Fermi energy. We added a line to the figure caption to clarify this point.

It would be good to emphasise more clearly some of the particular advances in this study over previous measurements by the authors and whether any reconsideration of this previous data should be in light of the new findings (https://doi.org/10.1016/j.cplett.2018.05.021)

The reviewer's point is well-taken. In the previous study we examined time independent absorption effects below, at, and above the absorption edge. In that study, there were no significant effects on the two-photon absorption cross section well below and above the edge, but there were strong resonance effects at the edge. The present study probes specifically the *σ** and *π** orbitals of the same system, while adding detailed calculations to enable a much richer understanding of the data. In particular, this new data suggests that in the first manuscript, the absorption at the edge involved significantly more complex physics than we were able to appreciate due to the quality of the underlying data. It is likely that with higher quality data, such as that shown here, the competing effects of saturable absorption and two-photon absorption would have been seen.

The readability of the figure labels and text could be improved by using a larger font. Figures also seem to be quite low resolution?

We apologize for the poor resolution, which may be due to PDF compression; we did not upload separate figures for the initial submission. We will make sure to address any readability issues should the paper be accepted by *JPCL*.

Editorial change requests:

1.) Please upload the Supporting Information (SI) as a separate file rather than including it in the manuscript file. Please use the "SI for Publication" file designation.

We have placed the SI into a separate file as requested.

2.) In both the main manuscript file and the Supporting Information, set the title in title case, with the first letter of each principal word capitalized.

We confirm that we have put the titles in the main manuscript and the SI in title case.

3.) Using acronyms in title is discouraged. Please spell out all acronyms in the title of the manuscript and Supporting Information.

We confirm that no acronyms are used in the title.

4.) Please label the abstract and shorten it to 150 words or fewer.

We labeled the abstract and shortened it from over 200 words to 150, in addition to making minor revisions to the text as outlined in the response to referees.

5.) Remove the section heading(s) throughout the body of the manuscript (you can leave a Methods heading).

We have removed the section headings in the body of the manuscript.

6.) In both the main file and the supporting information, fix the style of all references to use JPCL formatting (check all references carefully). ***JPC Letters reference formatting requires that journal references should contain: () around numbers, author names, article title (titles entirely in title case or entirely in lower case), abbreviated journal title (italicized), year (bolded), volume (italicized), and pages (first-last). Book references should contain author names, book title (in the same pattern), publisher, city, and year.

We changed the references to *JPCL* citation style using the .csl file linked on the website.

7.) URLs are not preferred references because website content can be modified and, consequently, the reference information may lack permanence (see References 2, 18, 19, and 26).

We confirm that no URLs are used as references (except insofar as DOI links are included consistent with *JPCL* citation style).

8.) Provide a TOC image per journal guidelines (2 in x 2 in; on the same page as the abstract) with the heading “TOC Graphic” above the graphic. The graphic should be in the form of a structure, graph, drawing, photograph, or scheme—or a combination. Non-scientific cartoon-like images or caricatures are discouraged. https://pubsapp.acs.org/paragonplus/submission/toc_abstract_graphics_guidelines.pdf

We have added a TOC graphic on the same page as the abstract and will also upload it in the portal.

9.) A brief, nonsentence description of the actual contents of each supporting information file is required. This description should be labeled Supporting Information and should appear before the Acknowledgement and Reference sections. Examples of sufficient and insufficient descriptions are as follows:
*Examples of sufficient descriptions: “Supporting Information: 1H NMR spectra for all compounds” or “Additional experimental details, materials, and methods, including photographs of experimental setup”.
*Examples of insufficient descriptions: “Supporting Information: Figures S1-S3” or “Additional figures as mentioned in the text”.

We have added a short non-sentence description of the SI before the Acknowledgments.

jz-2022-010204.R2

Name: Peer Review Information for "Saturable Absorption of Free-Electron Laser Radiation by Graphite Near the Carbon K-Edge"

Second Round of Reviewer Comments

Reviewer: 4

Comments to the Author

I have reviewed the revised manuscript and supplement, independently from other referees' reports and authors' response letter. Later on I have referred to the reports and response letter. I have not read the originally submitted version. In the present manuscript, the authors reported unusual behaviors on the measured transmitted intensity of a thin graphite film as a function of the FEL intensity, and characterized them to saturable absorption (SA) and two-photon absorption (TPA), with help of TDDFT simulations and a parameterized model expression. Since the present work extends well-known nonlinear processes of SA and TPA to the x-ray regime and potentially provides a comprehensive picture of the interaction of solid-state systems with high-intensity x-ray pulses, it is timely and will draw considerable attention from the community. However, the experimental data presented here have one weak point as they presented "transmitted intensity" rather than "transmission", which would make their findings less convincing. Moreover, the theoretical calculations do not support the experimental findings. Finally, the unusual behaviors in their measurement could be interpreted differently with simple estimates based on all experimental conditions, which I will discuss in details below. For these reasons, even though it has been throughout one round of the review process, I would like to ask major changes to the authors, taking into account the following points, before it is considered to be published in J. Phys. Chem. Lett.

1.

In Figs. 2a,b, the y axis should not be called "transmission". It is completely misleading. This was also pointed out by the second referee, but modifications by the authors were not sufficient. The statement of "We point out that Figs. 2a,b plot the transmitted X-ray intensity while Figs. 2c,d plot the fraction of transmitted and incoming intensity. Consequently a linear trend in Figs. 2a,b corresponds to a constant trend in Figs. 2c,d" should come much earlier. Alternatively, why don't the authors consider to plot "transmission" in Figs. 2a,b? Even though FEL intensity in the x axis was not calibrated (I remember this because of the response letter, but I'm not sure whether it is sufficiently clear from the manuscript itself), the straight lines made by the authors in Figs. 2a,b already imply that the transmission could be calculated by using the x axis, i.e., the (uncalibrated) FEL intensity. The problems of the current representation are: 1) In many places, the lines in Figs. 2a,b are referred as the transmission from the linear response, which is supposed to be "constant" as shown in many other literatures including [11]. This is very confusing. 2) It looks like that the increasing line in Figs. 2a,b matches well with the increasing trend in the corresponding region in Figs. 2c,d, which is of course completely misleading. They

are not the same quantities. The increasing line in Figs. 2a,b indicates the linear response, while the increasing trend in Figs. 2c,d represents the nonlinear response. To make a long story short, the two different representations in Figs. 2a,b and c,d confuse the readers drastically, and should be changed to be consistent.

Having said that, I realize it's rather critical to have calibration for incoming FEL intensity. Without such calibration, we cannot make the straight line in Figs. 2a,b in principle and we cannot define sub-linear and super-linear from there. How was the pulse energy varied? Was there any beam transmission loss as the intensity increases? In the previous work [11], the transmission was actually plotted, so how was the number of incident photons to the sample determined in that case? It would be great if the authors comment in the manuscript on why the calibration was not performed and what the implication for the conclusion would be.

2.

It will be useful to make an explicit comparison with the previous work [11], which was also pointed out by the third referee. In particular, regarding x-ray beam parameters, the photon energy was chosen slightly below the K edge (284.18 eV) in the previous work, whereas it is slightly above the K edge (285.7 eV) in the current work. Why did the author choose this photon energy and why do both of photon energies show enhancement of absorption, i.e., TPA-dominance? If TPA is related to resonance, then do both photon energies correspond to different resonances or one resonance? What was the energy bandwidth used in experiment (not mentioned)? Another noticeable difference from the previous work is the peak intensity used in the current work, which is an order of magnitude higher than the previous work. Why does it need higher intensity to show the absorption enhancement at the photon energy (285.7 eV) slightly above the K edge? I'm wondering this because Fig. S2 suggests that the resonant excitation cross sections at both photon energies are similar to each other, so the same behavior at the same intensity would be anticipated. If possible, it might be useful to estimate the TPA cross section in the current work and compare with the value in the previous work.

3.

TDDFT calculations and the expression parameterized with TDDFT results demonstrate that SA and TPA features are shown together, depending on the peak intensity. The next question is, can we explain why the π^* and σ^* transitions show each of these features? I don't see any theoretical support to interpret experimental observations. Do theoretical calculations reproduce experimental results? I'm doubtful for that, with the following arguments.

TDDFT calculations predict that the transmission is strongly enhanced from 10^11 W/cm^2 and then suppressed after 10^14 W/cm^2, i.e., both features are shown individually as a function of intensity. On the other hand, the experimental data demonstrated either enhanced or suppressed for each transition. Do the TDDFT results help to explain why? Are the fitting results (Table S1) useful? In fact, Table S1 says that the TPA contribution β of σ^* is 3 times larger than that of π^* and the SA contribution α_0 of σ^* is 2 times smaller than that of π^* (it's written reversely in the lines of 48-49). These are totally opposite to experimental findings.

In Fig. 2c, why do the TDDFT results have fluctuations, which potentially affect parameter fitting to the model?

In Figs. 3a,b, there are a sudden increase in absorption of σ^* for the two highest intensities, which seems to happen earlier than the π^* case (only the highest intensity). This increase in absorption may be related to TPA. However, the TPA feature was observed in experiment only for the π^* transition, but not for the σ^* transition. These figures also show the opposite trend to the experimental data.

So far, I have argued that the present TDDFT results neither reproduce nor help to interpret the experimental

findings. However, the authors need to seriously take into account the applicability of the TDDFT method to describe x-ray multiphoton physics (see the Auger decay treatment below), which was extensively pointed out by the second referee.

One more point is that not only a temporal pulse envelope as pointed out by the third referee, but a spatial fluence distribution profile also matters in theoretical calculations, so-called volume integration, if it is desirable to make a quantitative comparison with experimental data. This is not taken into account in the present theoretical consideration.

4.

I don't understand the statement of the lines of 51-54: "Auger decay events--and any sequential absorption processes following the formation of the resulting valence hole--are ... That said, Auger decay is unlikely to have played a significant role in these experiments due to the narrow spectral width and high spectral stability of the FEL source tuned into resonance with the 1s to π^* and 1s to σ^* transitions." This is the response to the second referee's point, but I don't think it's properly addressed at all. The second referee already gave possible scenarios; I would like to add a few words with simple estimates relevant to the current work.

First of all, I would like to argue that Auger decay has the most important role in x-ray multiphoton physics, in particular, for the given pulse duration and the given intensity regime of the current work. After resonant excitation from 1s to π^* (or σ^*), a 1s hole is formed. It will undergo possibly four different pathways. 1) The excited π^* electron is ionized by absorbing another photon. This corresponds to the resonant-enhanced two-photon process described by Fig. 1c. 2) An electron in the π or σ band is ionized by absorbing another photon. 3) The 1s hole is relaxed via Auger decay. If the Auger lifetime is shorter than the pulse duration, this process happens most likely. 4) It's also possible to make another resonant excitation from 1s at another atom (if it happens at the same atom, then it creates a double-core hole, but its transition energy will be quite off from the given photon energy). The authors argued that their x-ray beam was precisely tuned to resonance with a narrow bandwidth (the value is not given, though), so the processes of 1) and 2) are much less likely than 4). Then let's compare 3) Auger decay and 4) resonant excitation. The intensity applied in the current work was about 1-3x10^13 W/cm^2, which gave the highest flux of 6.5x10^14 ph/cm^2 fs. For the argument sake, let's put an optimistic estimate for the resonant excitation cross section of 100 Mb (a typical cross section of C in this photon energy is ~1 Mb). Then, the resonant excitation rate at the peak is 0.065 fs^-1, whereas the Auger rate is the inverse of the Auger lifetime (7 fs): 1/7=0.14 fs^-1, which is about two times larger than the resonant excitation rate. In other words, Auger decay still beats all relevant photoabsorption processes here, so it is most likely that Auger decay dominantly happens under the given experimental condition.

After Auger decay happens, it will create two valence holes in the π and/or σ bands. It will influence the electronic structure, and most likely the K edge (also the 1s transition energies) will be shifted to higher energhies. This could give a chance to make off-resonance, and thus to suppress absorption for both π^* and σ^* cases. On the other hand, the holes in the π and/or σ bands open up new resonance channels right below the π^* edge, as similar to the hidden resonance described in [30]. Combining with the blueshift mentioned in the above, the new resonance channels could give a chance to enhance absorption for the 285.7 eV case (then it becomes π or σ transition, rather than π^* transition). These are all hypotheses, inspired by the second referee, but they are alternative ways to explain suppression and enhancement of "transmission" in Figs. 2a,b.

I think these alternative scenarios are more feasible, given the fact that the FEL intensity used here is relatively low and it's hard to believe such low intensity reaches the saturation point to deplete the ground states. Unfortunately, the authors did not provide any

justification for this saturation point. Even though it is doubtful for applicability of TDDFT results, the fitted saturation intensity listed in Table S1 is about 10^16 W/cm^2, which is three orders of magnitude higher than the intensity regime under consideration. Note that all these physical processes mentioned in the above are readily included in a rate-equation approach, which has been widely used for gas-phase XFEL studies, for instances [28,29,30] and especially for resonant excitation [Ho et al., Phys. Rev. Lett. 113, 253001 (2014); Toyota et al., Phys. Rev. A 95, 043412 (2017); Rudek et al., Nat. Commun. 9, 4200 (2018)].

Reviewer: 3

Comments to the Author

Thank you for the authors for their response to the comments. I am happy with the modification to the figure caption.

X-ray Spectral Characterization

My experience at working at the C K edge at FERMI is that that a significant proportion of the laser shots have profiles that are far from Gaussian (though I agree it is marked improvement on SASE output). As the authors state that the spectral shape and bandwidth is crucial for these measurements, it would be helpful, rather than including the reference, to include some spectral information from the PADRES spectrometer or, information on data filtering on the spectral quality if non-Gaussian shots have been rejected and excluded from the analysis. It would also be helpful for the authors to comment more specifically on the energy bandwidth requirements etc. for the physical phenomena i.e. does the FEL bandwidth need to be narrower than the core-hole lifetime broadening? In their response to reviewer 2, there is a comment on the central wavelength jitter from the FEL, so it would be good to see this claim supported by the experimental evidence as well as a comment on the energy resolution of the measurements. The paper cited does not really address the wavelength region of operation during these experiments.

Author's Response to Peer Review Comments:

See attached letter.

Author reply for manuscript jz-2022-010204.R1 by Hoffmann et al. "Saturable Absorption of Free-Electron Laser Radiation by Graphite Near the Carbon K-Edge"

Here we reply to Reviewers 3-4. We believe we have adequately addressed their comments as regards to our manuscript and have taken advantage of the opportunity to improve the quality of our arguments and the text. We have reproduced each of the reviewers' comments in black italics and provide our detailed replies in blue for clarity, along with the details of any responsive changes made to the manuscript. Other minor changes, principally improvements to the wording, have also been made to the manuscript. All significant changes in the manuscript and SI are marked with a yellow background and have been reproduced here for consistency.

We wish to thank the referees for providing valuable feedback.

Reviewer 3

Thank you for the authors for their response to the comments. I am happy with the modification to the figure caption.

We appreciate the positive feedback.

X-ray Spectral Characterization

My experience at working at the C K edge at FERMI is that that a significant proportion of the laser shots have profiles that are far from Gaussian (though I agree it is marked improvement on SASE output). As the authors state that the spectral shape and bandwidth is crucial for these measurements, it would be helpful, rather than including the reference, to include some spectral information from the PADRES spectrometer or, information on data filtering on the spectral quality if non-Gaussian shots have been rejected and excluded from the analysis. It would also be helpful for the authors to comment more specifically on the energy bandwidth requirements etc. for the physical phenomena i.e. does the FEL bandwidth need to be narrower than the core-hole lifetime broadening? In their response to reviewer 2, there is a comment on the central wavelength jitter from the FEL, so it would be good to see this claim supported by the experimental evidence as well as a comment on the energy resolution of the measurements. The paper cited does not really address the wavelength region of operation during these experiments.

We thank the reviewer for the detailed comment. We have also observed that many FEL shots have non-Gaussian beam profiles. In order to maintain a larger data set, we decided not to filter shots on the basis of beam mode. In a previous experiment, it was found that two-photon absorption is significantly less sensitive to the beam mode than other nonlinear processes. such as second-harmonic absorption.[11,25] The spectral bandwidth measured on the PADReS spectrometer is $9\cdot10^{-4}$ nm, and the wavelength jitter is negligible ($<10^{-4}$ nm). The corresponding spectral bandwidth at a photon energy of 285.7 eV is 0.06 eV, in the range of the core-hole lifetime broadening of 0.1 eV. (Hence the argument in our previous responses regarding being on resonance within the shot-to-shot fluctuations.) Based on a comparison of the core-hole lifetime with the FEL pulse duration and the timescales associated with possible Auger pathways. we

believe the arguments we presented in the previous revision remain valid. The additional characterization of a typical set of FEL shots that we provide below confirms that the beam parameters were indeed as described.

We added a section to the SI detailing the beam diagnostics together with a new 4-panel figure displaying a typical shot-to-shot series and corresponding statistics.

Addition to the SI:

At FERMI each individual FEL shot is characterized by the Photon Analysis Delivery and Reduction System (PADReS).[10] **Fig. S5** shows the shot-to-shot beam diagnostics for a representative dataset consisting of 443 FEL pulses. The FEL intensity was measured at two different positions. Typical intensity fluctuations are shown in **Fig. S5a**. The gas-based I_0 monitor at PADReS and an ellipsoidal beamline mirror drain current at the EIS-TIMEX beamline were used to calibrate the incoming FEL intensity (**Fig. S5b**). The PRESTO spectrometer was used to analyze the beam profile, wavelength, and spectral bandwidth. The central wavelength jitter is $<10^{-4}$ nm, and the spectral bandwidth is close to $9 \cdot 10^{-4}$ nm (0.06 eV at a photon energy of 285.7 eV) (**Fig. S5c,d**). We analyzed the beam profile but did not filter shots that were not perfectly Gaussian in order to maintain a larger data set. In a previous experiment, it was found that two-photon absorption is significantly less sensitive to the beam mode than other nonlinear processes, such as second-harmonic absorption.[11,12] **Fig. S5** presents shot-to-shot beam diagnostics for one dataset consisting of 443 FEL pulses.

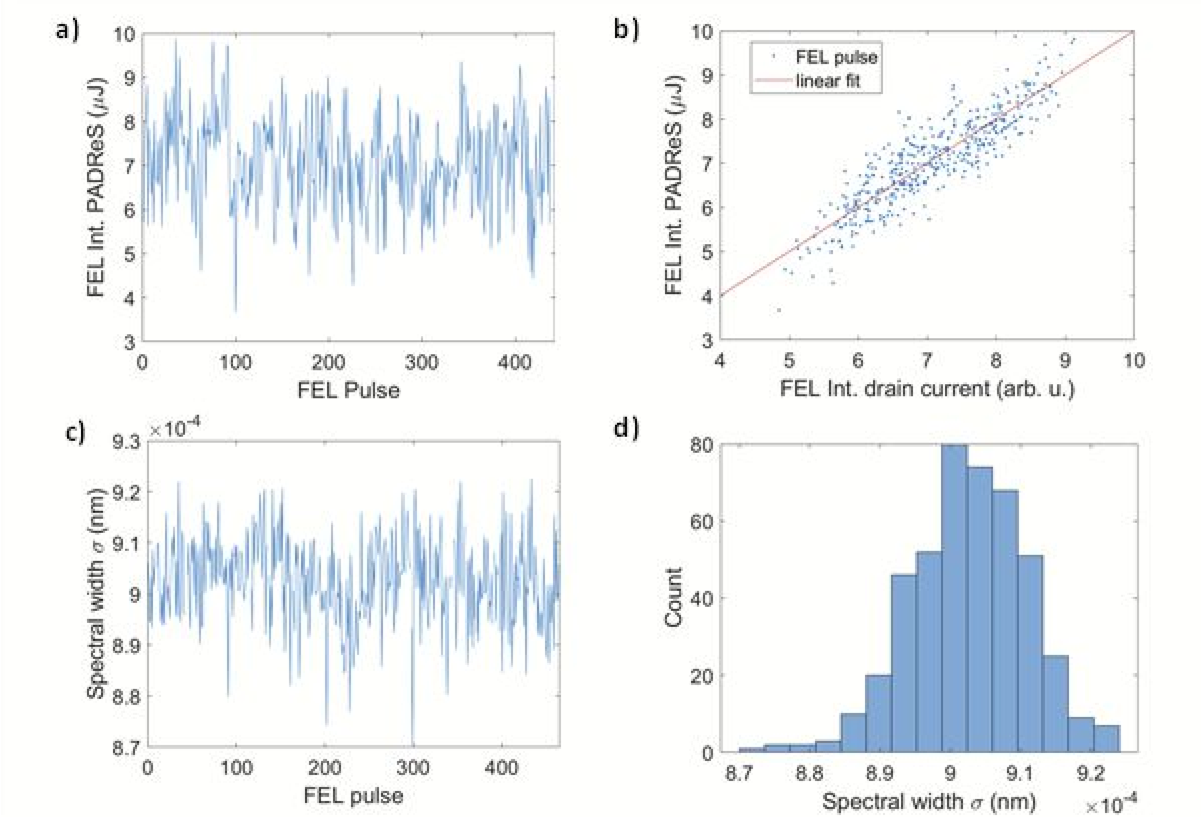

Figure S5: Shot-to-shot FEL beam diagnostics from PADReS. a) FEL pulse intensity variation for a typical set of FEL shots during the beamtime. b) FEL intensity calibration curve used for the analysis. c) spectral bandwidth and d) histogram of the spectral bandwidth as derived from PADReS beamline diagnostics.

We thank Reviewer 3 once again for their detailed report, helpful suggestions, and overall positive assessment of our work. We hope our latest changes mitigate all the concerns that remained.

Reviewer 4

I have reviewed the revised manuscript and supplement, independently from other referees' reports and authors' response letter. Later on I have referred to the reports and response letter. I have not read the originally submitted version. In the present manuscript, the authors reported unusual behaviors on the measured transmitted intensity of a thin graphite film as a function of the FEL intensity, and characterized them to saturable absorption (SA) and two-photon absorption (TPA), with help of TDDFT simulations and a parameterized model expression. Since the present work extends well-known nonlinear processes of SA and TPA to the x-ray regime and potentially provides a comprehensive picture of the interaction of solid-state systems with high-intensity x-ray pulses, it is timely and will draw considerable attention from the community. However, the experimental data presented here have one weak point as they presented "transmitted intensity" rather than "transmission", which would make their findings less convincing. Moreover, the theoretical calculations do not support the experimental findings. Finally, the unusual behaviors in their measurement could be interpreted differently with simple estimates based on all experimental conditions, which I will discuss in details below. For these reasons, even though it has been throughout one round of the review process, I would like to ask major changes to the authors, taking into account the following points, before it is considered to be published in J. Phys. Chem. Lett.

We appreciate the reviewer for taking the time to conduct this thorough review of all the materials at hand and to make additional suggestions, which we address in detail below. We are grateful for the overall positive assessment of the potential impact of our work.

1a) In Figs. 2a,b, the y axis should not be called "transmission". It is completely misleading. This was also pointed out by the second referee, but modifications by the authors were not sufficient. The statement of "We point out that Figs. 2a,b plot the transmitted X-ray intensity while Figs. 2c,d plot the fraction of transmitted and incoming intensity. Consequently a linear trend in Figs. 2a,b corresponds to a constant trend in Figs. 2c,d" should come much earlier.

We thank the reviewer for pointing out that the axis description can be misleading. We changed the axis description of Fig. 2a,b to make the distinction clearer within the caption. We also moved the sentence mentioned above to the first paragraph that contains Fig 2.

1b) Alternatively, why don't the authors consider to plot "transmission" in Figs. 2a,b? Even though FEL intensity in the x axis was not calibrated (I remember this because of the response letter, but

I'm not sure whether it is sufficiently clear from the manuscript itself), the straight lines made by the authors in Figs. 2a,b already imply that the transmission could be calculated by using the x axis, i.e., the (uncalibrated) FEL intensity. The problems of the current representation are: 1) In many places, the lines in Figs. 2a,b are referred as the transmission from the linear response, which is supposed to be "constant" as shown in many other literatures including [11]. This is very confusing. 2) It looks like that the increasing line in Figs. 2a,b matches well with the increasing trend in the corresponding region in Figs. 2c,d, which is of course completely misleading. They are not the same quantities. The increasing line in Figs. 2a,b indicates the linear response, while the increasing trend in Figs. 2c,d represents the nonlinear response. To make a long story short, the two different representations in Figs. 2a,b and c,d confuse the readers drastically, and should be changed to be consistent.

We thank the reviewer for the comment. Unfortunately, we cannot plot the experimental transmission in Fig. 2 panels a) and b) in the same way as the calculated transmission in panel c) and d), due to a lack of calibration allowing us to determine the absolute number of photons downstream of the sample. Therefore, we plot the transmitted intensity (uncalibrated) vs. incoming intensity obtained by the spectrometer CCD and I_0 monitor, respectively, in order to observe a trend between the two quantities. In order to make the representation clearer we changed the axis label and no longer call the experimental quantity that was measured “transmission”; we speak instead of “transmitted intensity.” As the reviewer pointed out, it should help that we reserve the term “transmission” for the ratio of outgoing to incoming intensity. The lines in Figs 2a,b represent a hypothetical linear response. from which the data deviates. By changing the axis description, the figure caption, and the terms used in the main text, we intend to make this distinction more pronounced.

1c) Having said that, I realize it's rather critical to have calibration for incoming FEL intensity. Without such calibration, we cannot make the straight line in Figs. 2a,b in principle and we cannot define sub-linear and super-linear from there. How was the pulse energy varied? Was there any beam transmission loss as the intensity increases? In the previous work [11], the transmission was actually plotted, so how was the number of incident photons to the sample determined in that case? It would be great if the authors comment in the manuscript on why the calibration was not performed and what the implication for the conclusion would be.

We thank the reviewer for the detailed comment. We do indeed have calibration for the incoming FEL intensity, but not for the transmitted intensity. As a result, we are unable to follow Ref. [11] and plot the absolute transmission. However, we can still observe whether the absorption follows a linear trend (i.e., the Beer–Lambert law) or whether it is nonlinear. In the latter case, we can also distinguish between super-linear and sub-linear absorption by a simple comparison with a linear fit. Not having the calibration means that we are unable to confirm the transmission percentage for the respective photon energy, though these values are widely available in the literature for the material studied here. In any case, we believe that this limitation does not render our observations of nonlinear effects or the wavelength-dependent trends invalid. To that end, we extended the above-mentioned sentences explaining **Fig. 2** that we moved to a section earlier in the manuscript. We hope this change improves the clarity of the text. Additional details on beam

diagnostics and sample data have also been provided in a new section of the SI, per our response to Reviewer 3 above.

*"We point out that **Figs. 2a,b** plot the transmitted X-ray intensity while **Figs. 2c,d** plot the transmitted intensity as a fraction of the incoming intensity. Consequently, a linear trend in **Figs. 2a,b** corresponds to a constant trend in **Figs 2c,d**. A more direct comparison of our calculated results with the experimental transmission would require calibration of the FEL intensity downstream of the sample, which is not available. The absence of this calibration neither limits our ability to observe nonlinear effects (by comparison with a linear fit) nor alters the trends we observed at each wavelength."*

2. It will be useful to make an explicit comparison with the previous work [11], which was also pointed out by the third referee. In particular, regarding x-ray beam parameters, the photon energy was chosen slightly below the K edge (284.18 eV) in the previous work, whereas it is slightly above the K edge (285.7 eV) in the current work. Why did the author choose this photon energy and why do both of photon energies show enhancement of absorption, i.e., TPA-dominance? If TPA is related to resonance, then do both photon energies correspond to different resonances or one resonance? What was the energy bandwidth used in experiment (not mentioned)? Another noticeable difference from the previous work is the peak intensity used in the current work, which is an order of magnitude higher than the previous work. Why does it need higher intensity to show the absorption enhancement at the photon energy (285.7 eV) slightly above the K edge? I'm wondering this because Fig. S2 suggests that the resonant excitation cross sections at both photon energies are similar to each other, so the same behavior at the same intensity would be anticipated. If possible, it might be useful to estimate the TPA cross section in the current work and compare with the value in the previous work.

The reviewer brings up an interesting point. In the previous work [11], the photon energy was chosen to be directly at the carbon K edge (284.18 eV), which is resonant with the π*. But choosing a photon energy directly at the edge can complicate things in the event that the band edge shifts due to the finite temporal length of the FEL pulse. In this work, we chose a slightly higher photon energy of 285.7 eV in order to be less sensitive to that effect while still in the same π* resonance. We added information about the FEL beam diagnostics to the SI, including the spectral bandwidth of 0.06 eV at 285.7 eV.

The larger peak intensity in this experiment compared to [11] stems from several differences between the experiments, including the beam being focused to a smaller size on the sample. With an approximate flux of $5*10^{29}$ photons/(cm^2*s), most of our FEL shots have intensities slightly larger than the intensities sampled in [11]. Another difference is that the samples used here are 80 nm thick compared to the 100 nm, 300 nm, and 500 nm thicknesses examined in Ref. [11]. We would expect a thinner sample to make the nonlinear effects more pronounced, especially when trying to observe saturable absorption. We agree that two-photon absorption may set in at lower intensities, but we do not have the necessary data points to locate the exact onset or to determine the two-photon absorption coefficient along the lines of [11]. In [11], the effect was observed at intensities an order of magnitude lower than the ones we measure here.

We added the following sentences to the main text to give a direct comparison to Ref. [11]:

"Several parameters of this experiment differ from previous measurements[11] and could lead to enhancement of saturable absorption. In particular, the sample used here was thinner, and the FEL spot size was smaller, which leads to larger measured intensities at similar pulse energies."

3a) TDDFT calculations and the expression parameterized with TDDFT results demonstrate that SA and TPA features are shown together, depending on the peak intensity. The next question is, can we explain why the π^* and σ^* transitions show each of these features? I don't see any theoretical support to interpret experimental observations. Do theoretical calculations reproduce experimental results? I'm doubtful for that, with the following arguments.

This appears to be a comment leading up to the points below. Please see detailed responses below.

3b) TDDFT calculations predict that the transmission is strongly enhanced from 10^11 W/cm^2 and then suppressed after 10^14 W/cm^2, i.e., both features are shown individually as a function of intensity. On the other hand, the experimental data demonstrated either enhanced or suppressed for each transition. Do the TDDFT results help to explain why? Are the fitting results (Table S1) useful? In fact, Table S1 says that the TPA contribution β of σ^* is 3 times larger than that of π^* and the SA contribution α_0 of σ^* is 2 times smaller than that of π^* (it's written reversely in the lines of 48-49). These are totally opposite to experimental findings.

First, we thank the reviewer for catching that mix-up in the text. The fitting results in Table S1 show that π* has a larger saturable absorption coefficient α_0. We corrected the statement in the manuscript. Second, as noted in our response to 1c, there are several factors that prevent a direct comparison between the simulated and measured spectrum, including sufficient data from our simulated spectrum in the energy range considered experimentally. Nevertheless, the simulations allows us to qualitatively understand the experimental results as resulting from SA.

3c) In Fig. 2c, why do the TDDFT results have fluctuations, which potentially affect parameter fitting to the model?

We thank the reviewer for the question regarding the TDDFT result. The fluctuation in *π** around ~10^{14} W/cm^2 is at the regime where the TPA process starts to kick in. At π* excitation, our TDDFT approach expects that the TPA contribution is not as pronounced as it is for the σ*. Consequently, the relaxation of excited electrons in this intensity range involves energies similar to the incident energy and generates photons of comparable wavelength, which manifests as the fluctuations. This issue might be resolved through the use of a denser intensity grid, but that would require additional simulations beyond the scope of the present work, given the substantial supercomputer time that would be required. We would like to point out once again that our approach is state of the art, and future work on the development of advanced and highly optimized codes will allow for improvements to these calculations. We added the following statement into the figure caption to address this issue.

“Fluctuations in 2c are due to competition between the TPA process and relaxation of excited electrons; the σ* does not exhibit this behavior due to a stronger TPA response. Gray dotted lines indicate the intensity range that was measured in the experiment. Note that a) and b) show the transmitted intensity while c) and d) show the transmitted intensity as a fraction of the incoming intensity.”

3d) In Figs. 3a,b, there are a sudden increase in absorption of σ^* for the two highest intensities, which seems to happen earlier than the π^* case (only the highest intensity). This increase in absorption may be related to TPA. However, the TPA feature was observed in experiment only for the π^* transition, but not for the σ^* transition. These figures also show the opposite trend to the experimental data.

The experiments do not extend to these higher intensities. We therefore respectfully disagree that the figures contradict the experimental data.

3d) So far, I have argued that the present TDDFT results neither reproduce nor help to interpret the experimental findings. However, the authors need to seriously take into account the applicability of the TDDFT method to describe x-ray multiphoton physics (see the Auger decay treatment below), which was extensively pointed out by the second referee.

This appears to be a guiding comment, for which we are grateful. We reply regarding these aspects in detail below.

3f) One more point is that not only a temporal pulse envelope as pointed out by the third referee, but a spatial fluence distribution profile also matters in theoretical calculations, so-called volume integration, if it is desirable to make a quantitative comparison with experimental data. This is not taken into account in the present theoretical consideration.

We thank the reviewer for the comment. The theoretical pulse envelope used in the simulation is designed to account for the cross-sectional area of the simulated unit cell, such that the total fluence and its spatial distribution correspond to that of the experiment. We added the following sentence to the computational details in the SI:

“To match the total fluence and its spatial distribution to the experiment, the pulse envelope is designed to account for the cross-sectional area of the simulated unit cell.”

4a) I don't understand the statement of the lines of 51-54: "Auger decay events--and any sequential absorption processes following the formation of the resulting valence hole--are ... That said, Auger decay is unlikely to have played a significant role in these experiments due to the narrow spectral width and high spectral stability of the FEL source tuned into resonance with the 1s to π^* and 1s to σ^* transitions." This is the response to the second referee's point, but I don't think it's properly addressed at all. The second referee already gave possible scenarios; I would like to add a few words with simple estimates relevant to the current work. First of all, I would like to argue that Auger decay has the most important role in x-ray multiphoton physics, in particular, for the given pulse duration and the given intensity regime of the current work. After resonant excitation from 1s to π^* (or σ^*), a 1s hole is formed. It will undergo possibly four different

pathways. 1) The excited π^* electron is ionized by absorbing another photon. This corresponds to the resonant-enhanced two-photon process described by Fig. 1c. 2) An electron in the π or σ band is ionized by absorbing another photon. 3) The 1s hole is relaxed via Auger decay. If the Auger lifetime is shorter than the pulse duration, this process happens most likely. 4) It's also possible to make another resonant excitation from 1s at another atom (if it happens at the same atom, then it creates a double-core hole, but its transition energy will be quite off from the given photon energy). The authors argued that their x-ray beam was precisely tuned to resonance with a narrow bandwidth (the value is not given, though), so the processes of 1) and 2) are much less likely than 4). Then let's compare 3) Auger decay and 4) resonant excitation. The intensity applied in the current work was about 1-3x10^13 W/cm^2, which gave the highest flux of 6.5x10^14 ph/cm^2 fs. For the argument sake, let's put an optimistic estimate for the resonant excitation cross section of 100 Mb (a typical cross section of C in this photon energy is ~1 Mb). Then, the resonant excitation rate at the peak is 0.065 fs^-1, whereas the Auger rate is the inverse of the Auger lifetime (7 fs): 1/7=0.14 fs^-1, which is about two times larger than the resonant excitation rate. In other words, Auger decay still beats all relevant photoabsorption processes here, so it is most likely that Auger decay dominantly happens under the given experimental condition.

We appreciate the reviewer's discussion on this point. Since no specific question is posed, we reply to these considerations below.

4b) After Auger decay happens, it will create two valence holes in the π and/or σ bands. It will influence the electronic structure, and most likely the K edge (also the 1s transition energies) will be shifted to higher energhies. This could give a chance to make off-resonance, and thus to suppress absorption for both π^* and σ^* cases. On the other hand, the holes in the π and/or σ bands open up new resonance channels right below the π^* edge, as similar to the hidden resonance described in [30]. Combining with the blueshift mentioned in the above, the new resonance channels could give a chance to enhance absorption for the 285.7 eV case (then it becomes π or σ transition, rather than π^* transition). These are all hypotheses, inspired by the second referee, but they are alternative ways to explain suppression and enhancement of "transmission" in Figs. 2a,b.

We thank the reviewer for this comment, which concerns alternative scenarios for explaining the experiments. As noted previously, while we cannot discount the possibility of Auger decay, we note that those effects, if prominent, would result in additional features in the linear absorption spectrum which were not observed in our experiments. Moreover, our calculations, which certainly do not include such physics, are consistent with the experiments. That said, stimulated by the insightful comments of this reviewer and the previous reviewer #2, we are currently developing the computational methodology to investigate the role of valence holes in the simulated spectrum, which will be the subject of a manuscript to follow.

We added the following sentence to the SI in appreciation of this discussion:

"Assessing the relative importance of additional decay channels discussed in the main text, such as Auger decay, will require further development of our computational methods."

4c) I think these alternative scenarios are more feasible, given the fact that the FEL intensity used here is relatively low and it's hard to believe such low intensity reaches the saturation point to deplete the ground states. Unfortunately, the authors did not provide any justification for this saturation point. Even though it is doubtful for applicability of TDDFT results, the fitted saturation intensity listed in Table S1 is about 10^16 W/cm^2, which is three orders of magnitude higher than the intensity regime under consideration. Note that all these physical processes mentioned in the above are readily included in a rate-equation approach, which has been widely used for gas-phase XFEL studies, for instances [28,29,30] and especially for resonant excitation [Ho et al., Phys. Rev. Lett. 113, 253001 (2014); Toyota et al., Phys. Rev. A 95, 043412 (2017); Rudek et al., Nat. Commun. 9, 4200 (2018)].

We thank the reviewer for this comment, but we respectfully disagree that the multi-photon physics observed here can be readily included in a rate-equation approach. One novel aspect of our current work is that it is (to our knowledge) the first to consider such effects in a solid-state sample. Previous works, including all those referenced by the reviewer, are based on XFEL studies of isolated atoms or molecules. The parameters required for the rate-equation approach (core-hole lifetimes, cross-sections, etc,) are currently unknown in this context. We agree that our study cannot answer all of the open mechanistic questions, and indeed we anticipate that our study will trigger wider interest in this topic, with additional experiments expanding the explored parameter space while theoreticians broaden the scope of the theory to account for additional decay channels. As noted above, we have already started work toward this end, but it will be reported in a new, different, manuscript.

We would like to take the opportunity to thank Reviewer 4 once again. Their detailed and insightful comments have helped us make significant improvements to the manuscript.

jz-2022-010204.R3

Name: Peer Review Information for "Saturable Absorption of Free-Electron Laser Radiation by Graphite Near the Carbon K-Edge"

Third Round of Reviewer Comments

Reviewer: 4

Comments to the Author

In my previous report, I raised three points: a) data representation, b) mismatch between theory and experiment, and c) alternative interpretation. Regarding a), the representation in the figures has been improved in the revised manuscript. Regarding c), I admit that it is a matter of scientific discussion and I appreciate their decision not to comment on this in their manuscript. On the other hand, the issue of b) still remains unsolved in the revised manuscript and my impression is that it won't be changed. Still I don't think the present experimental data support the theoretical results, or vice versa, but the authors disagreed with my assessment. Let me elaborate my understanding below.

The authors chose two photon energies: 285.7 eV for 1s→π^* and 309.2 eV for 1s→σ^*. From TDDFT simulations, the authors claimed that, for both photon energies, SA is dominant for low intensity and TPA is dominant for high intensity. For each photon energy, there is a transition from SA to TPA and its onset is around 10^14 W/cm^2. This is considered the main finding as stated in the abstract. Based on the parameterized expression, they also claimed that SA is dominant for 1s→π^* and TPA is dominant for 1s→σ^*.

The question is that, are these theoretical claims verified with their experiment? The answer is simply no, because the experimental data show only TPA for 1s→π^* and only SA for 1s→σ^*. This is clearly the opposite to their conclusion from the fitted parameters, which has been acknowledged by the authors. Furthermore, do the experimental measurements contain signatures of both SA and TPA for each transition? The answer is no. The intensity range in their measurement was not sufficient to observe both phenomena, and there was no hint for such observation in their experimental data. Thus, their experimental finding does not support any of their theoretical claims. Instead of discussing discrepancies, they just took advantage of TDDFT simulations in order to argue that in experiment TPA is for 1s→π^* because it was measured at higher intensities and SA is for 1s→σ^* because it was measured at lower intensities. In my view, this is not a validation of the TDDFT simulations. Is there any other evidence to validate TDDFT simulations in other SA/TPA experiments? No references were given in this direction. Then, how can one verify their main conclusion, which is solely from their simulations, "for lower intensities the nonlinear contribution to the absorption is dominated by SA attributed to ground-state depletion; for larger intensities (>10^14 W/cm^2), TPA becomes more dominant" (quoted from the abstract)? Even worse, the present experiment never reached to 10^14 W/cm^2. Therefore, the onset of 10^14 W/cm^2 is completely meaningless in their experiment.

Moreover, it becomes more mysterious if one compares with the previous experiment [11], where only TPA was found at even lower intensity. How is the present theoretical assertion applicable for the previous experiment? The argument of SA for low intensity and TPA for high intensity doesn't work here. All these observations make me wonder the validity of the theoretical results, thus placing the main conclusion of the present work questionable.

One more comment on 10^14 W/cm^2. To make a quantitative comparison and make the theoretical estimate of 10^14 more meaningful, it is necessary to take into account the fluence spatial distribution of the cross-sectional area, as newly stated in the revised supplement. However, the details are not given and it is difficult to understand how it's taken into account. For example, what is the shape of the experimental spatial envelope? How many fluence (or intensity) points were used to cover the experimental spatial envelope? The x-axis label in Fig. 2(c) and (d) shows intensity, but is it the peak intensity of the spatial envelope or is it one single value used for a theoretical calculation?

I originally thought that the present work provided a benchmark for nonlinear response of a solid sample to intense x-ray pulses, expecting to see a quantitative comparison between theory and experiment. However, after clarifying all the representation of figures and reading all the materials carefully, I'm more convinced that theoretical and experimental results are qualitatively different and they do not support each other. Therefore, I'm doubtful whether this work provides any significantly new insight or conceptual breakthrough towards our understanding of nonlinear interaction with intense x-rays, especially in view of the criteria of the Letter format. Based on all above considerations, I'm afraid that my final recommendation is not to publish this manuscript in J. Phys. Chem. Lett. Instead, I would strongly recommend to transfer it to a regular article in J. Phys. Chem. C.

Author's Response to Peer Review Comments:

Author reply for manuscript jz-2022-010204.R2 by Hoffmann et al. "Saturable Absorption of Free-Electron Laser Radiation by Graphite Near the Carbon K-Edge"

Here we reply to Reviewers 4. We believe we have adequately addressed their comments as regards to our manuscript and have taken advantage of the opportunity to improve the quality of our arguments and the text. We have reproduced each of the reviewers' comments in black italics and provide our detailed replies in blue for clarity, along with the details of any responsive changes made to the manuscript.

We wish to thank the referees for providing valuable feedback.

Reviewer 4

In my previous report, I raised three points: a) data representation, b) mismatch between theory and experiment, and c) alternative interpretation. Regarding a), the representation in the figures has been improved in the revised manuscript. Regarding c), I admit that it is a matter of scientific discussion and I appreciate their decision not to comment on this in their manuscript.

We are pleased to read that the data representation is now acceptable as well as our discussion of alternative explanations that we have incorporated in the last revision and SI.

On the other hand, the issue of b) still remains unsolved in the revised manuscript and my impression is that it won't be changed. Still I don't think the present experimental data support the theoretical results, or vice versa, but the authors disagreed with my assessment. Let me elaborate my understanding below.

We appreciate the time the reviewer took to provide additional discussion that is helpful in the overall discourse to which we reply below.

The authors chose two photon energies: 285.7 eV for 1s→π^* and 309.2 eV for 1s→σ^*. From TDDFT simulations, the authors claimed that, for both photon energies, SA is dominant for low intensity and TPA is dominant for high intensity. For each photon energy, there is a transition from SA to TPA and its onset is around 10^14 W/cm^2. This is considered the main finding as stated in the abstract. Based on the parameterized expression, they also claimed that SA is dominant for 1s→π^* and TPA is dominant for 1s→σ^*. The question is that, are these theoretical claims verified with their experiment? The answer is simply no, because the experimental data show only TPA for 1s→π^* and only SA for 1s→σ^*. This is clearly the opposite to their conclusion from the fitted parameters, which has been acknowledged by the authors. Furthermore, do the experimental measurements contain signatures of both SA and TPA for each transition? The answer is no. The intensity range in their measurement was not sufficient to observe both phenomena, and there was no hint for such observation in their experimental data. Thus, their experimental finding does not support any of their theoretical claims. Instead of discussing discrepancies, they just took advantage of TDDFT simulations in order to argue that in experiment TPA is for 1s→π^* because it was measured at higher intensities and SA is for 1s→σ^* because it was measured at lower intensities. In my view, this is not a validation of the TDDFT simulations. Is there any other evidence to validate TDDFT

simulations in other SA/TPA experiments? No references were given in this direction. Then, how can one verify their main conclusion, which is solely from their simulations, "for lower intensities the nonlinear contribution to the absorption is dominated by SA attributed to ground-state depletion; for larger intensities (>10^14 W/cm^2), TPA becomes more dominant" (quoted from the abstract)? Even worse, the present experiment never reached to 10^14 W/cm^2. Therefore, the onset of 10^14 W/cm^2 is completely meaningless in their experiment.

We appreciate the detailed discussion. Unpacking this argument, it appears that the main objection is that the intensities in the theory and experiment are different (with the saturation predicted to occur at much larger intensities in the theory). We recognized this, and thus modified the text to say "*Several parameters of this experiment differ from previous measurements*[11] *and could lead to enhancement of saturable absorption. In particular, the sample used here was thinner, and the FEL spot size was smaller, which leads to larger measured intensities at similar pulse energies.*" We believed that this adequately placed our findings in the correct context, however, we are willing to add an additional qualifying statement to the text (new additions highlighted in yellow):

"*Our calculations indicate that TPA will dominate for both photon energies at intensities greater than ~10^{14} W/cm^2. We expect that improvements in the simulation methodology, including a better description of the electron-hole screening and more extensive sampling of the ground state starting structure to include finite temperature effects, will lead to even better agreement in the predicted intensities, and will be explored in future works.*"

Furthermore, the sentence in the abstract "*By applying real-time electronic structure calculations, we find that for lower intensities the nonlinear contribution to the absorption is dominated by SA attributed to ground-state depletion; for larger intensities (>10^{14} W/cm^2), TPA becomes more dominant*." may have been hard to decipher due to the compact phrasing required by the word limit imposed by formatting requirements. The way we understand this sentence is that the TPA dominant regime is observed in the simulation that is constrained by the measurements that due to experimental limitations were conducted at slightly (Factor 2) lower intensities. Such intensities, while we could not reach them (which is not claimed), are reachable at other sources and hence we find value in exploring this parameter range; again, this is not arbitrary as the experimental data for two different transition matches closely at just slightly lower intensities. Repeating an essential sentence from the manuscript "*Our calculations indicate that TPA will dominate for both photon energies at intensities greater than ~10^{14} W/cm^2*" we clearly phrase that the insight stems from calculation and we use the word *indicate* to reflect being thoughtful about it.

We see two possible ways to address this. 1) We could crop all figures at, e.g., $5x10^{13}$ W/cm², i.e., the upper end of the measurement, and only speak of a SA/TPA mix regime at this point. Or 2) Show and discuss the regime that arises from slight extrapolation of the model that is otherwise clamped in the mid 10^{13} W/cm² regime.

We choose to do the latter as we do believe that there is no reason to entirely discount the model at a factor of two higher intensity. We on purpose use careful wording to reflect this circumstance. For the broader discussion, we think it is very helpful to show that our models predict a drastic change of regimes to motivate future experiments at different machines and/or careful consideration of interpreting spectra taken at high intensity in any material in the future. The primary motivation for this work is to evaluate how one can correctly interpret absorption spectra at extreme intensities as they are regularly used at free-electron lasers. Our work is a concert of experiment and theory. We appreciate the position the reviewer takes that a model/theory assumes the role to validate experimental data at each data point. There is nothing wrong with such a perspective in general, however it is not our intention in this work, and we purposely combine theory and experiment to gain broader insights and not just to validate the measurement. That is also reflected by shared corresponding authorship between experimental and theory groups and mixed first/second author between experiment and theory.

We like to note that we also describe that coherently in our summary paragraph: *"… Data collected at intensities of up to ~10^{13} W/cm^{2} exhibited a decrease or an increase in transmission with intensity, relative to a linear response, for the respective 1s to π* and 1s to σ* transitions. We attribute this behavior to different transition dipoles shifting the regime where TPA becomes dominant over SA at different threshold intensities. Our calculations indicate that TPA will dominate for both photon energies at intensities greater than ~10^{14} W/cm^{2}. Our experimental methods combined with theoretical calculations enable additional insights into nonlinear processes that occur due to the absorption of intense radiation at X-ray energies and can readily be extended to other materials. ...*"

To remedy the imperfections in the abstract that may stem from too compacted wording we could modify the one sentence to: "*By applying real-time electronic structure calculations, we find that for lower intensities the nonlinear contribution to the absorption is dominated by SA attributed to ground-state depletion; for larger intensities (>10^{14} W/cm^{2}), our model suggests TPA becomes more dominant*."

Moreover, it becomes more mysterious if one compares with the previous experiment [11], where only TPA was found at even lower intensity. How is the present theoretical assertion applicable for the previous experiment? The argument of SA for low intensity and TPA for high intensity doesn't work here. All these observations make me wonder the validity of the theoretical results, thus placing the main conclusion of the present work questionable.

We appreciate the concern but respectfully disagree. It is worth pointing out that there are several authors of Ref. 11 on this current manuscript and the differences between the work have been discussed in-depth before this manuscript was drafted. The consensus is that simply theory (not even DFT was used in Ref. 11) and data interpretation has improved in the last 4 years as it should. The data quality in our current experiment has sharply improved and we consider saturable absorption in addition to two-photon absorption as separate effects. We don't find this particularly concerning; it simply reflects progress on the matter at hand and our current experiment is different in nature and objective, hence a different work. In addition, in hindsight,

the choice of energy sitting right at the edge for Ref. 11 was particularly unfortunate, as it made the experiment sensitive to effects that are not present here. In this way the present experiment is simpler and easier to interpret, while being far from a repeat or increment of the experiment in Ref. 11.

One more comment on 10^14 W/cm^2. To make a quantitative comparison and make the theoretical estimate of 10^14 more meaningful, it is necessary to take into account the fluence spatial distribution of the cross-sectional area, as newly stated in the revised supplement. However, the details are not given and it is difficult to understand how it's taken into account. For example, what is the shape of the experimental spatial envelope? How many fluence (or intensity) points were used to cover the experimental spatial envelope? The x-axis label in Fig. 2(c) and (d) shows intensity, but is it the peak intensity of the spatial envelope or is it one single value used for a theoretical calculation?

We thank the reviewer for bringing up these additional questions. To clarify the approach more, we added to the Supplementary Information the following additional sentence: "The theoretical intensity is determined from applied electric field strength of the sinosoidal envelope. The total energy of the envelope over time matches the intensity in the experiment and the one reported in Fig 2c/2d."

I originally thought that the present work provided a benchmark for nonlinear response of a solid sample to intense x-ray pulses, expecting to see a quantitative comparison between theory and experiment. However, after clarifying all the representation of figures and reading all the materials carefully, I'm more convinced that theoretical and experimental results are qualitatively different and they do not support each other. Therefore, I'm doubtful whether this work provides any significantly new insight or conceptual breakthrough towards our understanding of nonlinear interaction with intense x-rays, especially in view of the criteria of the Letter format. Based on all above considerations, I'm afraid that my final recommendation is not to publish this manuscript in J. Phys. Chem. Lett. Instead, I would strongly recommend to transfer it to a regular article in J. Phys. Chem. C.

While we respectfully disagree with the reviewer in the overall assessment based on the responses and our understanding of the matter at hand, we thoroughly thank the reviewer for the very detailed response and discussion. The manuscript has become much improved due to their feedback.
